# BAFF-based trifunctional T-cell engagers trigger robust tumor immunity against B-cell malignancies

**DOI:** 10.1093/procel/pwaf054

**Published:** 2025-06-27

**Authors:** Shuhong Li, Licai Shi, Qiaoru Guo, Lijun Zhao, Xuexiu Qi, Zelin Liu, Zhi Guo, Yu J Cao

**Affiliations:** State Key Laboratory of Chemical Oncogenomics, Shenzhen Key Laboratory of Chemical Genomics, Peking University Shenzhen Graduate School, Shenzhen 518055, China; State Key Laboratory of Chemical Oncogenomics, Shenzhen Key Laboratory of Chemical Genomics, Peking University Shenzhen Graduate School, Shenzhen 518055, China; State Key Laboratory of Chemical Oncogenomics, Shenzhen Key Laboratory of Chemical Genomics, Peking University Shenzhen Graduate School, Shenzhen 518055, China; State Key Laboratory of Chemical Oncogenomics, Shenzhen Key Laboratory of Chemical Genomics, Peking University Shenzhen Graduate School, Shenzhen 518055, China; State Key Laboratory of Chemical Oncogenomics, Shenzhen Key Laboratory of Chemical Genomics, Peking University Shenzhen Graduate School, Shenzhen 518055, China; Department of Hematology, Affiliated Nanshan Hospital of Shenzhen University, Shenzhen 518052, China; Department of Hematology, Affiliated Nanshan Hospital of Shenzhen University, Shenzhen 518052, China; State Key Laboratory of Chemical Oncogenomics, Shenzhen Key Laboratory of Chemical Genomics, Peking University Shenzhen Graduate School, Shenzhen 518055, China; Institute of Chemical Biology, Shenzhen Bay Laboratory, Shenzhen 518132, China

**Keywords:** T-cell engager, natural ligand, multispecific antibody, B-cell malignancy, antibody engineering, immune escape

## Abstract

Advancements in protein engineering have driven the continuous optimization of T-cell engagers (TCEs), resulting in remarkable clinical outcomes in the treatment of B-cell malignancies. Moreover, developing tri- or multispecific TCEs has emerged as a promising strategy to address the challenges of tumor heterogeneity and antigen escape. However, considerable obstacles remain, primarily in format design. In this study, we engineered BAFF-based TCEs with various formats that incorporate anti-CD3 Fab or IgG domains fused with BAFF ligands to target BAFF receptors (BAFFR, BCMA, and TACI). These constructs varied in valency and the presence or absence of long-acting elements such as Fc domains or the albumin binding domain consensus sequence (ABDCon). Although the inclusion of an Fc domain did not enhance sustained tumor eradication, variations in valency and spatial configuration profoundly influenced cytotoxicity. We identified TriBAFF/CD3/ABDCon as the optimal trifunctional construct, featuring an anti-CD3 Fab backbone with BAFF and ABDCon fused to the C-termini of the heavy and light chains. This design facilitates optimal immune synapse formation between the target cells and T cells and effectively controls tumor burdens in various B-cell malignancy models with good tolerability. Notably, TriBAFF/CD3/ABDCon outperformed conventional therapies, including blinatumomab and BAFF-based CAR-T cells, in models of heterogeneous leukemia and aggressive lymphoma. These findings underscore the potential of using natural ligands as antibody-targeting modules and provide valuable insights into the design of the next generation of multispecific TCEs, which hold promise for improving treatment outcomes in a wide range of malignancies and beyond.

## Introduction

Treating B-cell malignancies is among the most promising areas in immunotherapy, with several targeted therapies, such as bispecific antibodies (bsAbs) and chimeric antigen receptor-T (CAR-T) cells, demonstrating significant clinical potential (Tapia-Galisteo et al., 2023; van de Donk and Zweegman, 2023; Zheng et al., 2024). In recent years, bsAbs have garnered increasing attention as a cancer treatment strategy because of their flexible and versatile mechanisms of action (Falchi et al., 2023; Labrijn et al., 2019). To date, 15 bsAbs have received regulatory approval, 10 of which are classified as T-cell engagers (TCEs) (Klein et al., 2024), underscoring the rapid advancement of this class of antibodies as a novel therapeutic modality. Moreover, CAR-T cell therapies have been established as powerful immunotherapy approaches, receiving approval for the third-line treatment of myeloma, lymphoma, and leukemia with expanded indications for second-line use in select cases (Cappell and Kochenderfer, 2023; Lu and Jiang, 2022). Despite its transformative potential, CAR-T cell therapy is still hampered by challenges such as toxicity, the need for chemotherapy pretreatment, stringent patient eligibility criteria, lengthy preparation times, and high costs, which collectively limit its accessibility to a broader patient population (Amini et al., 2022; Cappell and Kochenderfer, 2023; Trabolsi et al., 2024). In contrast, TCEs offer notable advantages, including milder side effects, off-the-shelf availability, multiple administration routes, and suitability for earlier lines of treatment, positioning TCEs as compelling and accessible alternatives to the CAR-T cell approach (Holstein et al., 2023; Trabolsi et al., 2024; Velasquez et al., 2018).

Despite the substantial progress achieved with TCE therapies, some patients with B-cell malignancies remain resistant to treatment, primarily due to tumor antigen heterogeneity and antigen escape, which continue to pose considerable challenges in this field (Al Hadidi et al., 2024; Falchi et al., 2023). A promising approach to overcoming these limitations lies in the development of next-generation TCEs capable of simultaneously targeting multiple tumor antigens (Liu et al., 2022; Zhang et al., 2019; Zhao et al., 2022, 2024). To this end, various multispecific TCEs have been developed (Ding et al., 2023; Gauthier et al., 2019; Liu et al., 2022; Seung et al., 2022; Wu et al., 2020; Zhao et al., 2022) and are typically categorized into two main groups: antibody fragments and IgG-like constructs, which vary in terms of size, valency, and interdomain configuration. However, designing multifunctional TCEs that effectively integrate and balance the functions of multiple targeting modules remains both critical and complex (Cao et al., 2021b; Cheung et al., 2019; Du et al., 2017; Liu et al., 2022; Zhang et al., 2019; Zhao et al., 2022). Owing to their inherent ability to recognize and bind to multiple markers on malignant cells, natural ligands are promising solutions to the above challenges and have been successfully utilized in therapies such as conventional CAR-T, chimeric autoantibody receptor (CAAR), and immunotoxin, capitalizing on their unique properties as antigen binding motifs (Ramírez-Chacón et al., 2022). Recently, our group demonstrated that natural ligands can serve as adaptors for universal CAR-T cells, enabling them to target multiple tumor antigens (Cao et al., 2021a; Li et al., 2024), thereby facilitating the rapid and switchable redirection of CAR-T cells across various tumor lineages. Building on this concept, we hypothesized that a similar strategy could be extended to increase the efficacy of multispecific TCE therapies. While prior studies have highlighted the impact of certain factors, such as molecular weight, antibody affinity, and valency, on the biodistribution and cytotoxicity of bispecific TCEs (Cheng et al., 2016; Seckinger et al., 2017; Zuch de Zafra et al., 2019), the structural dynamics of ligand-based constructs remain poorly understood, particularly in terms of how the native structures of the tumor necrosis factor (TNF) superfamily of ligands are preserved on the antibody backbone. Therefore, identifying and characterizing the key structural parameters that govern the potency and stability of ligand-based TCEs is essential for optimizing their therapeutic efficacy and enabling successful clinical translation.

In this study, we describe the development and characterization of several trifunctional TCE formats that utilize the B-cell activating factor (BAFF) ligand, which specifically targets multiple B-cell antigens, including BAFFR, BCMA, and TACI. The optimal construct, TriBAFF/CD3/ABDCon, was engineered by fusing the BAFF ligand and an albumin binding domain consensus sequence (ABDCon) to the C-terminus of the light and heavy chains of the anti-CD3 Fab (SP34). The natural homotrimerization of BAFF enables TriBAFF/CD3/ABDCon to adopt a trivalent format, preserving the natural conformation of the ligand while significantly enhancing T-cell-redirected efficacy and effectively extending its *in vivo* half-life (Jacobs et al., 2015; Zhang et al., 2021). Compared with IgG-like formats, TriBAFF/CD3/ABDCon facilitates the formation of an optimal immunological synapse and demonstrates dose-dependent antitumor potency both *in vitro* and *in vivo* with no off-target toxicity and excellent tolerability. Notably, this construct effectively overcomes CD19-mediated immune escape, exhibiting efficacy against heterogeneous leukemia that is superior to that blinatumomab, while also addressing the design limitations of natural ligands and outperforming conventional BAFF CAR-T cells in lymphoma treatment. Overall, we present the development of a distinctive, trifunctional, natural ligand-based TCE with a desirable structure that could mitigate the risk of immune escape and simplify the clinical transition of T-cell-redirected therapies.

## Results

### Preparation and characterization of Fab-structured BAFF-based TCEs

**Figure 1.**
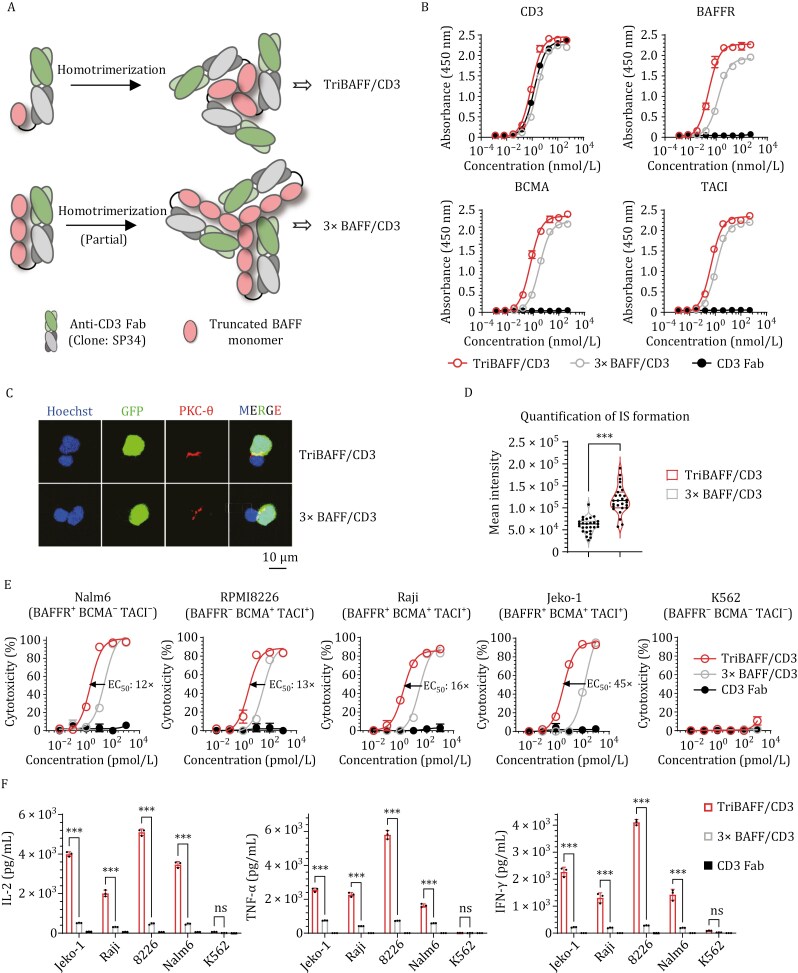
***In vitro*** comparison of Fab-structured BAFF-based TCEs with different trimeric configurations. (A) Schematic of the Fab-structured BAFF-based TCEs panel: TriBAFF/CD3 and 3× BAFF/CD3. The green and gray domains represent anti-human CD3 Fab (clone: SP34), and the pink domains represent the truncated BAFF ligand (amino acids 134–285). (B) Binding profiles of two Fab-structured BAFF-based TCEs to the antigens (CD3, BAFFR, BCMA, and TACI) determined by ELISA. (C) Representative confocal images of the IS from three independent experiments. Human T cells were cocultured with Nalm6-GFP cells in the presence of 1 nmol/L BAFF-based TCE for 1 h, and cell–cell conjugates were imaged at 100× oil objective magnification using a laser scanning confocal microscope (Nikon, A1R). Hoechst (blue), anti-PKC-θ (red), GFP (green), and merged images of all the stains are shown. Scale bar = 10 μm. (D) Statistical analysis of the mean fluorescence intensity of PKC-θ at the IS in (C). *P* values were determined by paired two-tailed *t*-tests. (E) Cytotoxicity assays of different Fab-structured BAFF-based TCEs were performed with T cells against the indicated target cells at an E:T ratio of 2:1 for 24 h. The results shown are from one of two independent experiments. (F) Inflammatory cytokine release assays. Human T cells, along with 1 nmol/L corresponding Fab-structured BAFF-based TCEs or CD3 Fab, were cocultured with specific target cells for 24 h at an E:T ratio of 1:1. Two-way ANOVA with multiple comparisons in Dunnett correction were used to assess the significance. Error bars represent mean ± SD. *P < 0.05, **P < 0.01, and ***P < 0.001; ns indicates not significant (*P* ≥ 0.05).

The BAFF ligand was chosen as the antigen-targeting module because of its trispecificity, which endows the antibodies with trivalent, multi-indication targeting capabilities. We began our study of BAFF-based TCEs based on the bispecific T-cell engager (BiTE), a bsAb format that is widely employed in T-cell redirection applications and has the same format as the FDA-approved blinatumomab. Given the critical importance of the trimeric configuration of the BAFF ligand (Ullah and Mackay, 2023), which is a member of the TNF family, one of our primary design principles was ensuring the stability of this trimeric configuration. For this purpose, we conjugated truncated BAFF ligands (Li et al., 2021; Wong et al., 2022) to the C-terminus of either the monomeric or tandem trimeric form (Li et al., 2021; Schmidts et al., 2019) of anti-CD3 Fab (Fig. 1A). Both Fab-structured BAFF-based TCEs were prepared via standard mammalian expression and affinity purification. SDS-PAGE analysis revealed that both TriBAFF/CD3 and 3× BAFF predominantly existed as homotrimers; however, TriBAFF/CD3 exhibited superior homogeneity compared to its counterpart (Fig. S1A). Furthermore, under accelerated degradation conditions, TriBAFF/CD3 demonstrated markedly enhanced protein stability relative to the 3× BAFF format (Fig. S2A and S2B). The binding affinities of both antibody formats were assessed via flow-based cell binding assays and ELISA-based antigen binding, both of which gave consistent results (Figs. 1B and S3A). TriBAFF/CD3 demonstrated significantly stronger binding to BAFFR/BCMA/TACI (or tumor cells) than did 3× BAFF/CD3. With respect to CD3 (or T-cell) interactions, TriBAFF/CD3 exhibited greater affinity than did CD3 Fab, whereas 3× BAFF/CD3 showed reduced affinity. We attributed these observations to the enhanced stability conferred by the trivalent structure of TriBAFF/CD3. The formation of immune synapse (IS) mediated by antibodies between tumor cells and T cells is a crucial indicator of structural optimization. Protein kinase C-θ (PKC-θ) is essential for T-cell activation and serves as a marker of the extent of IS formation. In accordance with the binding affinities, TriBAFF/CD3 yielded the formation of more ISs than did 3× BAFF/CD3 (Fig. 1C and 1D). Additionally, TriBAFF/CD3 promoted the expression of surface molecules associated with T-cell activation, including CD69 and CD25 (Fig. S3B). To assess the potency of both formats, we performed *in vitro* cytotoxicity assays in which the Fab-structured BAFF-based TCEs engaged T cells against various B-cell malignancy cell lines. As expected, TriBAFF/CD3 demonstrated significantly enhanced cytotoxicity, exhibiting a greater than 10-fold increase in sensitivity (improving the EC_50_ by 12- to 45-fold) compared with 3× BAFF/CD3 (Fig. 1E). The significant increase in cytokine release further confirmed these differences (Fig. 1F). These findings suggest that the spontaneously formed TriBAFF/CD3 homotrimer significantly enhanced structural stability, homogeneity, and antitumor activity *in vitro*, indicating that this stable trimeric conformation could be the key to developing BAFF-based TCEs. Thus, the TriBAFF/CD3 antibody format is the optimal Fab-structured BAFF-based TCE.

### Development of trifunctional Fab-structured BAFF-based TCEs

We subsequently modified the pharmacokinetic profile of TriBAFF/CD3 by incorporating a serum albumin (SA) binding peptide (ABDCon) into the optimal Fab-structured BAFF-based TCE format (Fig. S4A). ABDCon, a phage-displayed short peptide variant, was designed using consensus sequence design (Jacobs et al., 2015; Zhang et al., 2021). Notably, ABDCon has a high affinity for albumin from various species, including mice, rats, and cynomolgus, thereby facilitating preclinical, toxicological, and pharmacological studies and allows it to serve as an effective scaffold for extending the half-life of antibodies (Tan et al., 2021). Analytical size-exclusion chromatography (SEC) was used to assess the structural homogeneity of both homotrimeric configurations (Fig. S4B). The TriBAFF/CD3 conjugate containing the ABDCon peptide (TriBAFF/CD3/ABDCon) retains a relatively high affinity for human SA (HSA) and a relatively low affinity for mouse SA (MSA), as reported (Fig. S4C). TriBAFF/CD3/ABDCon exhibited a slightly reduced antigen binding capacity, which was attributed to the increase in steric hindrance resulting from the incorporation of the short peptide (Fig. S4D). This attenuation was similarly observed in cytotoxicity and cytokine release assays (Fig. S5A and S5B). However, no significant differences were observed in the extent of IS formation or the proportion of activated T cells between the two groups (Fig. S5C–E). Notably, *in vitro* assays demonstrated that neither Fab-structured BAFF-based TCE induced significant tumor cell proliferation (Fig. S6). In summary, integrating ABDCon maintained the overall potency of the optimal Fab-structured BAFF-based TCE, giving rise to trifunctional antibodies that target T cells and tumor cells and are involved in SA/FcRn cycling.

### Development of trifunctional IgG-structured BAFF-based TCEs

**Figure 2.**
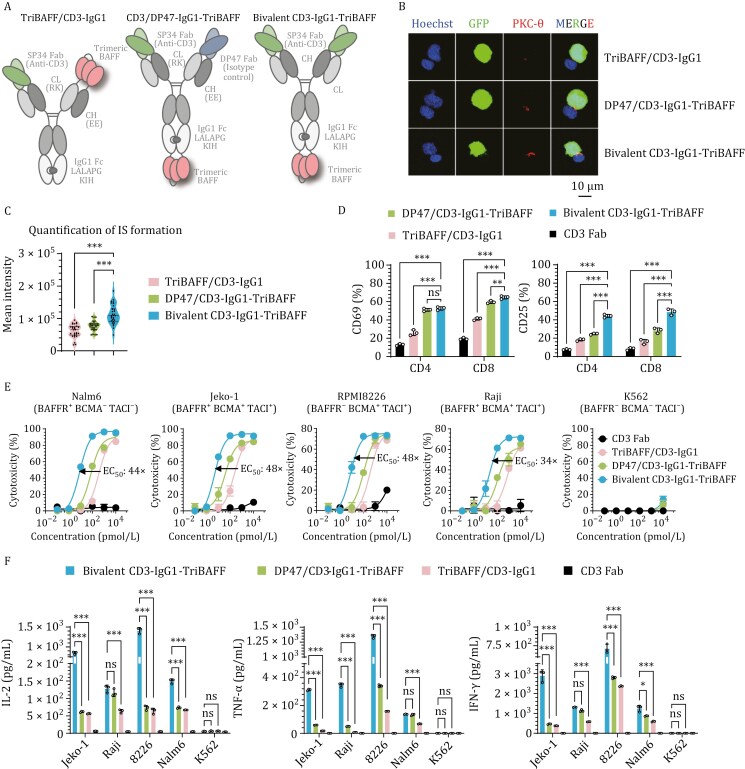
***In vitro*** comparison of different IgG-structured BAFF-based TCEs. (A) Schematic of the IgG-structured BAFF-based TCEs panel: TriBAFF/CD3-IgG1, DP47/CD3-IgG1-TriBAFF, and Bivalent CD3-IgG1-TriBAFF. The green and gray domains represent anti-human CD3 Fab (clone: SP34), the blue domains represent the non-CD3 binder DP47 (isotype control), and the pink domains represent the truncated BAFF ligand (amino acids 134–285). (B) Representative confocal images of IS from three independent experiments. Human T cells were cocultured with Nalm6-GFP cells in the presence of 1 nmol/L BAFF-based TCE for 1 h, and cell–cell conjugates were imaged at 100× oil objective magnification using a laser scanning confocal microscope (Nikon, A1R). Hoechst (blue), anti-PKC-θ (red), GFP (green), and merged images of all the stains are shown. Scale bar = 10 μm. (C) Statistical analysis of the mean fluorescence intensity of PKC-θ at the IS in (B). *P* values were determined by paired two-tailed *t*-tests. (D) Left: Frequency of CD69^+^ T cells after 20 h of coculture with Nalm6 cells. Right: Frequency of CD25^+^ T cells after 96 h of coculture with Nalm6 cells. The assays included CD3 Fab as a control. Two-way ANOVA multiple comparisons in Dunnett correction were used to assess the significance. (E) Cytotoxicity assays of different IgG-structured BAFF-based TCEs were performed with T cells against the indicated target cells at an E:T ratio of 2:1 for 24 h. The results shown are from one of two independent experiments. (F) Inflammatory cytokine release assays. Human T cells, along with 1 nmol/L corresponding IgG-structured BAFF-based TCEs or CD3 Fab, were cocultured with specific target cells for 24 h at an E:T ratio of 1:1. Two-way ANOVA multiple comparisons in Dunnett correction were used to assess the significance. Error bars represent mean ± SD. *P < 0.05, **P < 0.01, and ***P < 0.001; ns indicates not significant (*P* ≥ 0.05).

Next, we engineered BAFF-based TCEs with an IgG heterodimer, another antibody format that is widely utilized in T-cell redirection applications. We generated three IgG1-like antibodies, each integrating trimeric BAFF in a 2 + 1 configuration with monovalent or bivalent T-cell binding, as increasing the valence state of CD3 has been reported to potentially enhance antitumor efficacy (Santich et al., 2020) (Fig. 2A). For the constructs with monovalent CD3 Fab, we employed the CrossMab strategy (Regula et al., 2018) to prevent chain mismatch and enhance protein product homogeneity (Fig. S1B). Based on our previous findings that exposure of the C-terminus of BAFF is critical for antigen binding (Li et al., 2024), we positioned trimeric BAFF at either the N- or C-terminus of the IgG1 antibody backbone. As expected, blocking the C-terminus of the BAFF trimer significantly reduced the antigen binding capacity of the antibody TriBAFF/CD3-IgG1, whereas bivalent display of CD3 Fab (bivalent CD3-IgG1-TriBAFF) markedly enhanced the T-cell binding capacity (Fig. S7A and S7B). Furthermore, the trends in the extents of IS formation and T-cell activation were comparable (Fig. 2B–D), confirming our hypothesis. Subsequent cytotoxicity and cytokine release assays further confirmed that these differences contribute to the antitumor potency of IgG-structured BAFF-based TCEs, with an increase in sensitivity of more than 30-fold (ranging from 34- to 48-fold) (Fig. 2E and 2F). Taken together, these results indicate the following: (i) monovalent CD3 Fab is inadequate to elicit robust antitumor effects and (ii) exposure of the BAFF C-terminus is essential for BAFF-based antibody activity. The superiority of the bivalent CD3-IgG1-TriBAFF construct underscores that the steric configuration and binding valence should be optimized for IgG1-like antibodies to elicit the most potent *in vitro* antitumor response.

### Optimized spatial configuration and valency improve tumor and T cell binding of trifunctional BAFF-based TCEs

**Figure 3.**
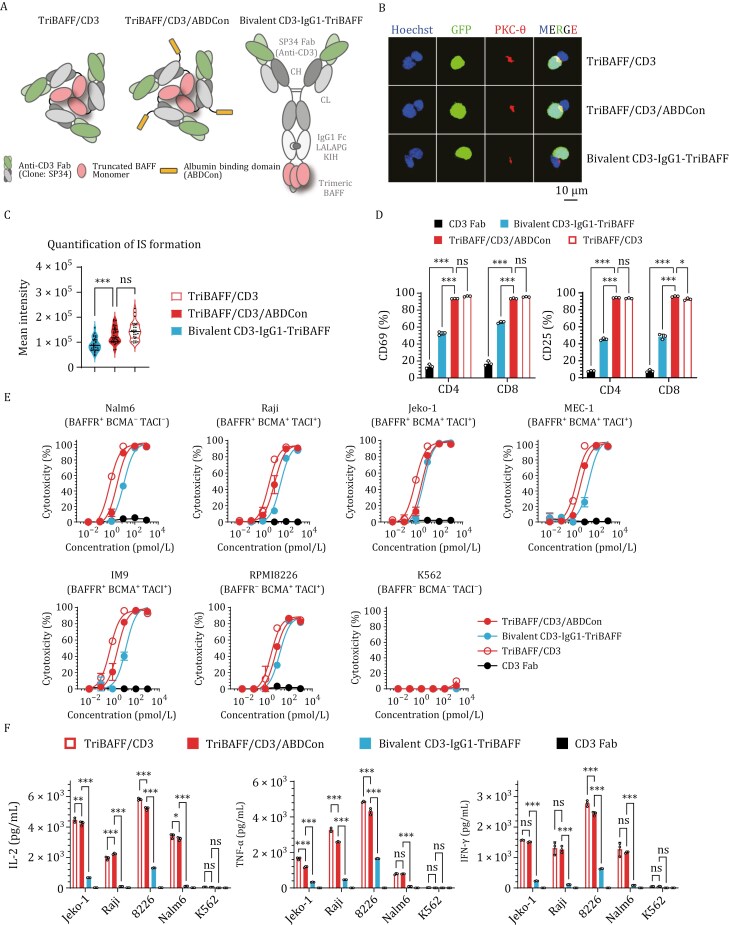
***In vitro*** comparison of the Fab-structured and IgG-structured BAFF-based TCEs. (A) Schematic of the optimal Fab-structured and IgG-structured BAFF-based TCEs panel: TriBAFF/CD3, TriBAFF/CD3/ABDCon, and Bivalent CD3-IgG1-TriBAFF. The green and gray domains represent anti-human CD3 Fab (clone: SP34), the yellow domains represent albumin binding peptide (ABDCon), and the pink domains represent truncated BAFF ligands (amino acids 134–285). (B) Representative confocal images of IS from three independent experiments. Human T cells were cocultured with Nalm6-GFP cells in the presence of 1 nmol/L BAFF-based TCE for 1 h, and cell–cell conjugates were imaged at 100× oil objective magnification using a laser scanning confocal microscope (Nikon, A1R). Hoechst (blue), anti-PKC-θ (red), GFP (green), and merged images of all the stains are shown. Scale bar = 10 μm. (C) Statistical analysis of the mean fluorescence intensity of PKC-θ at the IS in (B). *P* values were determined by paired two-tailed *t*-tests. (D) Left: Frequency of CD69^+^ T cells after 20 h of coculture with Nalm6 cells. Right: Frequency of CD25^+^ T cells after 96 h of coculture with Nalm6 cells. The assays included CD3 Fab as a control. Two-way ANOVA multiple comparisons in Dunnett correction were used to assess the significance. (E) Cytotoxicity assays of different BAFF-based TCEs were performed with T cells against the indicated target cells at an E:T ratio of 2:1 for 24 h. The results shown are from one of two independent experiments. (F) Inflammatory cytokine release assays. Human T cells, along with 1 nmol/L corresponding BAFF-based TCEs or CD3 Fab were cocultured with specific target cells for 24 h at an E:T ratio of 1:1. Two-way ANOVA multiple comparisons in Dunnett correction were used to assess the significance. Error bars represent mean ± SD. *P < 0.05, **P < 0.01, and ***P < 0.001; ns indicates not significant (*P* ≥ 0.05).

Based on our previous exploration, we identified the optimal configurations of Fab- and IgG- structured BAFF-based TCEs, followed by a comparative analysis of these two configurations (Fig. 3A). Among the generated constructs, those with optimal configurations demonstrated potent antitumor efficacy while maintaining straightforward structural designs and achieving high expression yields (Table S1). We then initiated our comparative analyses by determining the antigen binding affinity of all the constructs via ELISA but did not observe a significant difference in the antigen binding capacity of the two formats (Fig. S8A). Nonetheless, TriBAFF/CD3/ABDCon demonstrated significantly greater efficacy than Bivalent CD3-IgG1-TriBAFF in both the intensity of IS formation and proportion of T cells activated (Fig. 3B–D), which may be due to the CD3 Fab valence state. As expected, TriBAFF/CD3/ABDCon induced enhanced tumor cell lysis and cytokine release, demonstrating significant superiority compared with Bivalent CD3-IgG1-TriBAFF (Fig. 3E and 3F). Similarly, TriBAFF/CD3/ABDCon exhibited significantly enhanced cytotoxicity and cytokine release than Bivalent CD3-IgG1-TriBAFF across a range of cells derived from patients with B-cell malignancies (Fig. S8B and S8C). We hypothesized that the homotrimeric format resulted in improved potency, while appropriate interdomain spacing, specifically the length of one CH1 chain rather than the length of CH1–CH2–CH3, facilitated binding among different targets. Moreover, the homotrimeric configuration of TriBAFF/CD3 and TriBAFF/CD3/ABDCon enhances antitumor efficacy by enabling optimized trivalent CD3 engagement, a critical determinant in the design of potent multispecific TCE therapeutics. These findings further underscore the efficacy of the structural optimization process and the clinical potential of the optimized configuration.

Previously, we developed a split-design BAFF-based sCAR-T strategy for ligand-guided targeting (Li et al., 2024), whereas TriBAFF/CD3/ABDCon represents a fully optimized, antibody-based TCE with a distinct mechanistic approach. We next compared the functional performance of these two modalities. Cytotoxicity assays revealed comparable tumor cell killing between TriBAFF/CD3/ABDCon and Myc-BAFF-based sCAR-T cells; however, TriBAFF/CD3/ABDCon showed slightly reduced activity at low concentrations (Fig. S9A). This may be attributed to steric hindrance caused by the N-terminal fusion of the CD3 Fab heavy chain in TriBAFF/CD3/ABDCon, whereas the Myc-BAFF format may allow more efficient immunological synapse formation between tumor and sCAR-T cells. Similar trends were observed in cytokine release assays, with the sCAR-T approach exhibiting slightly higher cytokine secretion (Fig. S9B).

In terms of pharmacokinetics, TriBAFF/CD3/ABDCon and Bivalent CD3-IgG1-TriBAFF exhibited comparable serum half-lives of approximately 80–90 h. In contrast, TriBAFF/CD3 was unable to bind to SA and was gradually cleared from the circulation after 15 h (Fig. S10). Collectively, these results suggest that the homotrimeric BAFF configuration and bivalency of CD3 Fab contribute to the enhanced antitumor activity of BAFF-based TCEs. Furthermore, TriBAFF/CD3/ABDCon is a trifunctional TCE that displayed the best T cell and tumor cell targeting effects among all of the candidate constructs, with the prolonging half-life *in vitro*.

### Antitumor effects of the trifunctional BAFF-based TCEs *in vivo*

**Figure 4.**
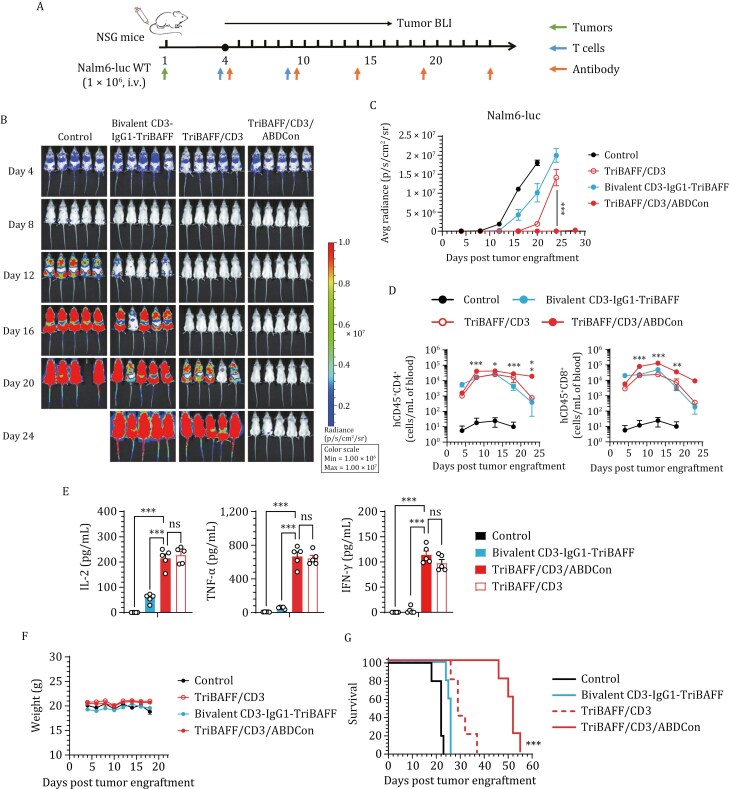
***In vivo*** comparison of the Fab-structured and IgG-structured BAFF-based TCEs. (A) Timeline of the *in vivo* experiments. Consistent results were obtained from two independent experiments (*n* = 5 mice). (B) Representative bioluminescence images of mice subjected to different treatments. The colors represent the luminescence intensity (red, highest; blue, lowest). (C) Quantification of the average luminescence intensity (p/s/cm^2^/sr). Two-way ANOVA multiple comparisons in Dunnett correction were used to assess the significance, comparing TriBAFF/CD3/ABDCon with TriBAFF/CD3. (D) Assessment of the presence of persistent human CD4^+^ (hCD45^+^CD4^+^, left) and CD8^+^ (hCD45^+^CD8^+^, right) T cells in the peripheral blood by flow cytometry over a 3-week follow-up period. Two-way ANOVA multiple comparisons in Dunnett correction were used to assess the significance, comparing TriBAFF/CD3/ABDCon with Bivalent CD3-IgG1-TriBAFF at each time point. (E) Evaluation of serum inflammatory cytokine release by ELISA 2 h after TCE administration. One-way ANOVA multiple comparisons in Dunnett correction were used to assess the significance. (F) Changes in the body weights of the mice during TCE treatment. (G) Survival curves of the mice subjected to the indicated treatments compared using the log-rank (Mantel–Cox) test. Error bars represent mean ± SEM.*P < 0.05, **P < 0.01, and ***P < 0.001; ns indicates not significant (*P* ≥ 0.05).

To assess how the valency and spatial configuration of the CD3-specific antibody affect the tumor response *in vivo*, we evaluated candidates with various antibody configurations in the Nalm6 xenograft model. As illustrated in Fig. 4A, NSG mice bearing human acute lymphoblastic leukemia (ALL) tumors (Nalm6, B-ALL) were treated with human T cells alongside various BAFF-based TCEs. As expected, TriBAFF/CD3/ABDCon exhibited the most potent antitumor activity, significantly outperforming Bivalent CD3-IgG1-TriBAFF, which is consistent with the *in vitro* functional study results. Although treatment with TriBAFF/CD3 initially elicited a response comparable to that of TriBAFF/CD3/ABDCon, tumor recurrence rapidly occurred because of the short half-life and limited durability of TriBAFF/CD3 (Fig. 4B and 4C). TriBAFF/CD3/ABDCon induced enhanced T-cell expansion and prolonged T-cell persistence, with a more pronounced effect on the CD4 subtype (Fig. 4D). Furthermore, TriBAFF/CD3/ABDCon exhibited more potent induction of cytokine release than Bivalent CD3-IgG1-TriBAFF did, but was not significantly different compared to TriBAFF/CD3 (Fig. 4E). Regarding safety, mice treated with various BAFF-based TCEs presented no indication of toxicity, such as weight loss or alopecia (Fig. 4F). As expected, TriBAFF/CD3/ABDCon significantly prolonged the survival of tumor-bearing mice compared with the other treatments (Fig. 4G). Collectively, these results align with our *in vitro* findings and emphasize the importance of optimizing the valency of CD3-specific antibodies, utilizing BAFF homotrimeric structures, and prolonging the half-life.

### Sensitivity and safety of the trifunctional BAFF-based TCEs

**Figure 5.**
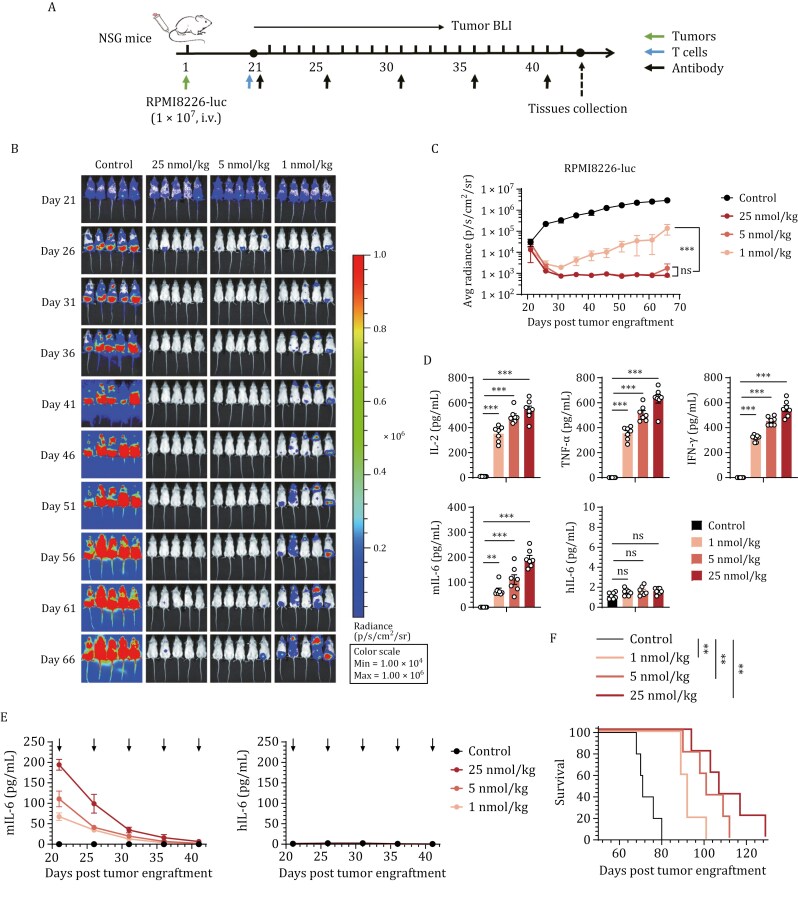
Sensitivity and safety of the trifunctional BAFF-based TCEs ***in vivo***. (A) Timeline of the *in vivo* experiments. Consistent results were obtained from two independent experiments (*n* = 5 mice). (B) Representative bioluminescence images of mice subjected to different treatments. The colors represent the luminescence intensity (red, highest; blue, lowest). (C) Quantification of the average luminescence intensity (p/s/cm^2^/sr). Two-way ANOVA multiple comparisons in Dunnett correction were used to assess the significance, comparing the indicated treatments. (D) Evaluation of serum inflammatory cytokine release by ELISA 2 h after TCE administration. One-way ANOVA multiple comparisons in Dunnett correction were used to assess the significance. (E) Evaluation of serum murine and human IL-6 release by ELISA 2 h after each administration of TriBAFF/CD3/ABDCon. (F) Survival curves of the mice subjected to the indicated treatments compared using the log-rank (Mantel–Cox) test. Error bars represent mean ± SEM. *P < 0.05, **P < 0.01, and ***P < 0.001; ns indicates not significant (*P* ≥ 0.05).

A MM xenograft model (RPMI8226) was subsequently used to assess the *in vivo* therapeutic sensitivity and safety of the optimal candidate construct. As shown in Fig. 5, three weeks after RPMI8226 tumor transplantation, the mice were administered human T cells along with various dosages (1, 5, or 25 nmol/kg) of TriBAFF/CD3/ABDCon. Pronounced therapeutic sensitivity was evidenced by the comparable tumor regression observed upon administration of the low to high doses of TriBAFF/CD3/ABDCon at the initiation of therapy (Fig. 5B and 5C). Over time, tumor recurrence was noted in the low-dose group, whereas complete remission persisted in the middle- and high-dose groups until day 66 (Fig. 5B and 5C). Two h following the initial administration of TriBAFF/CD3/ABDCon, the cytokine levels in the peripheral blood of the mice were positively correlated with the administered dosage (Fig. 5D). Although all the mice treated with TriBAFF/CD3/ABDCon exhibited transient weight loss and a modest increase in monocyte-derived murine IL-6 levels (which decreased throughout the experiment), no significant increase in the human IL-6 level was observed (Fig. 5E). This was likely due to the ABDCon peptide binding to MSA, which triggered the T-cell-mediated activation of murine myeloid cells. Overall, no significant indicators of toxicity, such as sustained weight loss, alopecia, sudden mortality, or vital tissue damage, were observed in the mice treated with either the high or low doses of TriBAFF/CD3/ABDCon (Fig. S11A and S11B). This further validated the safety and efficacy of trivalent CD3 in TriBAFF/CD3/ABDCon. Ultimately, the survival duration of the mice in the high-, middle-, and low-dose groups was significantly prolonged compared with that in the control group (Fig. 5F). Overall, the trifunctional TCE TriBAFF/CD3/ABDCon demonstrates not only robust therapeutic sensitivity *in vivo* but also a good safety profile.

### The BAFF-based TCE overcomes CD19 immune escape *in vitro* and *in vivo*

One approach to address the issues of antigen loss and emergence of tumor escape variants following successful CD19-targeting therapy is to target other cell surface molecules, such as BAFF-R, BCMA, or TACI. We performed CRISPR CD19 gene knockout (KO) in two human B-cell tumor cell lines, Burkitt lymphoma (Raji) and ALL (Nalm6), to model disease relapse associated with CD19 loss. We subsequently assessed the cytotoxicity of human T cells induced by blinatumomab or TriBAFF/CD3/ABDCon against WT and CD19-KO tumor cells *in vitro*. Notably, blinatumomab was toxic to WT tumor cells only, whereas TriBAFF/CD3/ABDCon exhibited consistent toxicity to both WT and CD19-KO tumor cells, a conclusion further substantiated by the cytokine release data (Fig. S12A and S12B). These findings suggest that the optimized trifunctional BAFF-based TCE TriBAFF/CD3/ABDCon could effectively overcome immune escape. In contrast, in other WT cell lines, including MM, mantle cell lymphoma (MCL), and chronic lymphocytic leukemia (CLL) cell lines, TriBAFF/CD3/ABDCon exhibited inferior cytotoxicity compared with blinatumomab (Fig. S12A), which was potentially due to the significantly lower expression levels of BAFF-R, BCMA, and TACI in these cells relative to those of CD19.

**Figure 6.**
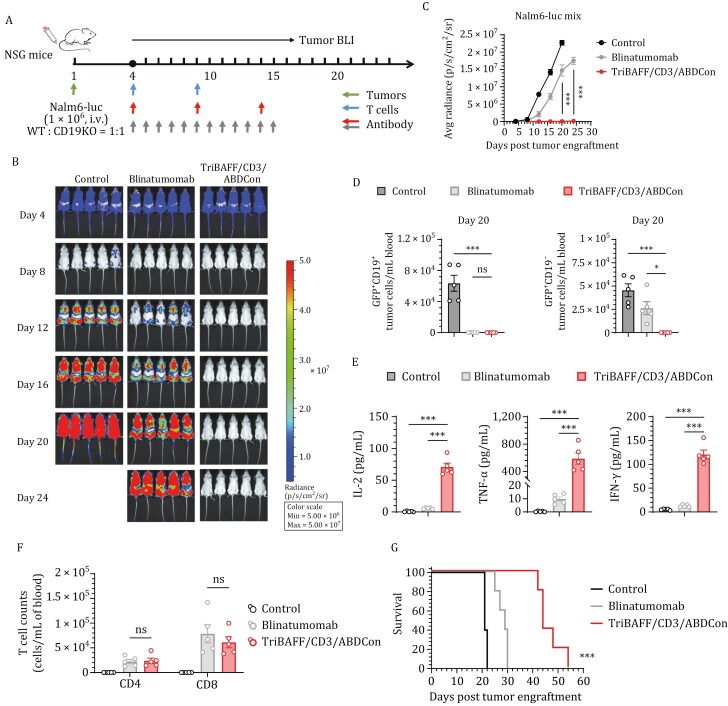
The BAFF-based TCE overcomes CD19 immune escape ***in vitro.*** (A) Timeline of the *in vivo* experiments. The red triangles represent TriBAFF/CD3/ABDCon administration, and the gray triangles represent blinatumomab administration. Consistent results were obtained from two independent experiments (*n* = 5 mice). (B) Representative bioluminescence images of mice subjected to different treatments. The colors represent the luminescence intensity (red, highest; blue, lowest). (C) Quantification of the average luminescence intensity (p/s/cm^2^/sr). Two-way ANOVA multiple comparisons in Dunnett correction were used to assess the significance, comparing TriBAFF/CD3/ABDCon with blinatumomab. (D) Assessment of the presence of tumor cells (GFP^+^CD19^+^ or GFP^+^CD19^−^) in the peripheral blood via flow cytometry on the 20th day of the experiment. One-way ANOVA multiple comparisons in Dunnett correction were used to assess the significance. (E) Evaluation of serum inflammatory cytokine release by ELISA 2 h after TCE administration. One-way ANOVA multiple comparisons in Dunnett correction were used to assess the significance. (F) On the 13th day of the experiment, the presence of persistent human CD3^+^ (hCD3^+^) T cells in the peripheral blood was assessed by flow cytometry. One-way ANOVA multiple comparisons in Dunnett correction were used to assess the significance. (G) Survival curves of the mice subjected to the indicated treatments compared using the log-rank (Mantel–Cox) test. Error bars represent mean ± SEM. *P < 0.05, **P < 0.01, and ***P < 0.001; ns indicates not significant (*P* ≥ 0.05).

We established a human B-ALL CD19 immune escape xenograft model using Nalm6 cells in NSG mice to assess how the therapeutic efficacy of TriBAFF/CD3/ABDCon compares with that of blinatumomab. Given that the half-life of blinatumomab is a mere 2–3 h, blinatumomab was administered daily. Human T cells were infused on days 4 and 9 following tumor inoculation, and the corresponding antibodies were administered via intraperitoneal injection in accordance with the experimental protocol outlined in Fig. 6A. Both TriBAFF/CD3/ABDCon and blinatumomab effectively eradicated established tumors during the initial stage of treatment (Fig. 6B). However, the tumors in the mice treated with blinatumomab recurred rapidly due to the emergence of CD19-KO tumor variants, whereas the tumors were controlled in the mice that received TriBAFF/CD3/ABDCon until day 24 (Fig. 6B and 6C). Flow cytometry confirmed that only TriBAFF/CD3/ABDCon eliminated both tumor populations by day 20 of the experiment, whereas blinatumomab treatment was ineffective because of the emergence of CD19-KO tumors (Figs. 6D and S13A). Furthermore, flow cytometry analysis of antigen expression in tumor cells from mice treated with TriBAFF/CD3/ABDCon revealed that BAFF-R was slightly downregulated (Fig. S13B), which may be attributable to the incomplete metabolism of TriBAFF/CD3/ABDCon in these mice. A residual amount of protein remained bound to the tumor surface, obstructing antibody binding. Similarly, the cytokine levels induced by TriBAFF/CD3/ABDCon were significantly higher than those elicited by blinatumomab two h after the initial administration (Fig. 6E), which might be attributable to the more potent initial response provoked by the high doses of TriBAFF/CD3/ABDCon administered at extended intervals. Nevertheless, TriBAFF/CD3/ABDCon was not superior in terms of T-cell expansion or persistence (Fig. 6F). Moreover, no significant weight loss was observed in the mice during the treatment (Fig. S13C). Ultimately, the survival duration of the mice treated with TriBAFF/CD3/ABDCon was significantly longer than that of the mice treated with blinatumomab (Fig. 6G).

To provide comparative validation against the FDA-approved CD19-targeted CAR-T therapy Tisagenlecleucel (Tisa-cel) for addressing CD19 antigen escape mechanisms, we generated CD19 CAR-T cells structurally consistent with Tisa-cel for subsequent validation. Consistent with the comparation results for Blinatumomab, TriBAFF/CD3/ABDCon demonstrated superior activity, particularly in CD19-negative and immune escape variant-containing mixed cell lines (Fig. S14A), as validated through cytokine release assays (Fig. S14B). We then established a CD19 heterogeneous B-ALL model to assess the *in vivo* activity differences between TriBAFF/CD3/ABDCon and CD19 CAR-T cells (Fig. S15A). Although mice treated with CD19 CAR-T cells initially showed reduced tumor burden compared to control mice, the tumors rapidly relapsed due to the emergence of Nalm6 cell variants lacking CD19 expression (Fig. S15B). In contrast, mice treated with TriBAFF/CD3/ABDCon exhibited sustained tumor control (Fig. S15C). Furthermore, TriBAFF/CD3/ABDCon triggered rapid T-cell cytokine release (2 h post-injection) that maintained tumor suppression with superior serum cytokine production compared to CD19 CAR-T cells (Fig. S15D). Finally, TriBAFF/CD3/ABDCon-treated mice exhibited prolonged survival versus CD19 CAR-T group, without significant treatment-related weight loss (Fig. S15E and S15F).

In conclusion, TriBAFF/CD3/ABDCon effectively overcomes CD19 immune escape both *in vitro* and *in vivo* via its distinctive, trifunctional, ligand-based TCE therapeutic strategy.

### The efficacy of BAFF-based TCE therapy is greater than that of conventional BAFF-based CAR-T cell therapy

Currently, BAFF-based CAR-T cell therapy is one of the most promising BAFF-based therapeutic modalities in clinical cancer research (Wong et al., 2022), but there have been no reports on BAFF-based antibody therapies. A phase I clinical trial of BAFF CAR-T cells (LMY-920, NCT05312801) is currently in progress (Tagami et al., 2024). Our previous research demonstrated that immobilizing BAFF on the surface of T cells compromises the receptor binding activity of BAFF CAR-T cells, as the necessary homotrimer configuration and spatial orientation are not maintained. Therefore, in this study, we compared the TriBAFF/CD3/ABDCon TCE with BAFF CAR-T cells incorporating the same tumor antigen binding domain (Wong et al., 2022). We engineered a BAFF-based CAR by utilizing a second-generation CAR backbone and employed lentiviral vectors to deliver the CAR gene into human T cells (Fig. S16A and S16B). The cytotoxicity induced by TriBAFF/CD3/ABDCon and BAFF CAR-T cells was assessed *in vitro*, and significantly greater toxicity to various cell lines, including ALL, CLL, MCL, Burkitt lymphoma, and MM cell lines, was observed after TriBAFF/CD3/ABDCon treatment (Fig. S16C). This conclusion is further supported by the cytokine release data (Fig. S16D). Thus, our hypothesis regarding the TCE platform was further validated.

**Figure 7.**
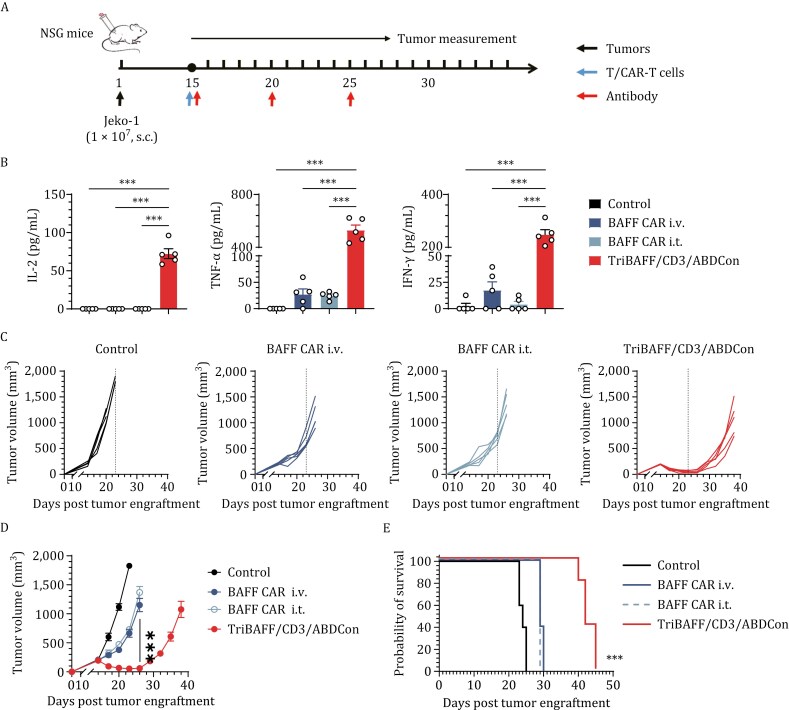
Enhanced efficacy of TriBAFF/CD3/ABDCon compared to BAFF-based conventional CAR T-cell therapy ***in vivo***. (A) Timeline of the *in vivo* experiments. A total of 3 × 10^7^ human T cells or BAFF CAR-T cells were intravenously administered on day 15, and 4 × 10^6^ BAFF CAR-T cells were intratumorally administered on day 15. Consistent results were obtained from two independent experiments (*n* = 5 mice). (B) Evaluation of serum inflammatory cytokine release by ELISA 2 h after administration. One-way ANOVA multiple comparisons in Dunnett correction were used to assess the significance. (C and D) Tumor size was monitored over 38 days. The tumor volume (mm^3^) was calculated using the formula (length × width^2^)/2. (E) Survival curves of the mice subjected to the indicated treatments compared using the log-rank (Mantel–Cox) test. Error bars represent means ± SEM. *P < 0.05, **P < 0.01, and ***P < 0.001; ns indicates not significant (*P* ≥ 0.05).

Then, we performed *in vivo* experiments. NSG mice bearing subcutaneous MCL tumors (Jeko-1) were administered BAFF CAR-T cells either intravenously or intratumorally, and human T cells were infused intravenously on day 15 after tumor inoculation (Fig. 7A). Two hours post-administration, TriBAFF/CD3/ABDCon induced potent cytokine release, which was significantly greater than that induced by BAFF CAR-T cells administered intravenously and intratumorally (Fig. 7B). TriBAFF/CD3/ABDCon also exhibited sustained antitumor activity, controlling the tumor burden without progression until day 25 (Fig. 7C and 7D). However, in the later phases of the experiment, the tumor surveillance capability of TriBAFF/CD3/ABDCon was lost due to T-cell exhaustion and antigen downregulation. Peripheral blood analysis revealed T-cell dynamics in TriBAFF/CD3/ABDCon-treated mice, with an initial proliferative surge followed by contraction kinetics concomitant with elevated PD-1 co-expression profiles (Fig. S17A and S17B). Similarly, tumor-infiltrating T cells exhibited an exhausted phenotype (Fig. S17C). Furthermore, flow cytometry showed downregulation of BAFFR and BCMA antigens, along with complete loss of TACI antigen in treated Jeko-1 tumors (Fig. S17D). In contrast, BAFF CAR-T cells exhibited weak antitumor activity initially, followed by rapid disease progression. As expected, mice treated with TriBAFF/CD3/ABDCon demonstrated significantly prolonged survival compared with those in the other treatment groups (Fig. 7E). Furthermore, none of the mice in any of the groups experienced weight loss during the treatment period, indicating that the drugs were well tolerated (Fig. S17E). In summary, the structurally optimized trifunctional BAFF-based TCE TriBAFF/CD3/ABDCon demonstrated overall efficacy that was superior to that of BAFF CAR-T cell therapy and represents a promising approach for ligand-based immunotherapy.

## Discussion

Trispecific or multispecific TCEs represent a promising strategy to overcome the challenges of antigen heterogeneity and escape in B-cell malignancies (Chen et al., 2024; Tapia-Galisteo et al., 2023; Wu et al., 2020; Zhao et al., 2022). While most recent efforts have focused on optimizing antibody combinations (Lou and Cao, 2022), we chose to modify the natural ligand BAFF for TCE therapy because of its ability to bind multiple tumor antigens (Tagami et al., 2024; Ullah and Mackay, 2023). This approach helps alleviate the time-consuming and labor-intensive process of developing structurally optimized combinations of multiple antibodies. Notably, this work provides a comprehensive analysis of the design, preparation, optimization and functional characterization of trifunctional BAFF-based TCEs and offers valuable insights into designing a new generation of trifunctional TCEs.

BAFF (also known as TNFSF13B or CD257) is a critical factor for B cell survival and a member of the TNF superfamily (Eslami and Schneider, 2021) that plays key roles in regulating B-cell homeostasis and immune responses. The BAFF receptors BAFFR, BCMA, and TACI are widely expressed in various leukemia, lymphoma, and myeloma cell subtypes (Ullah and Mackay, 2023). While BAFF-based targeted therapies, such as conventional or switchable CAR-T cells and toxin fusion systems, have been extensively explored (Lyu et al., 2007; Nilforoushzadeh et al., 2024; Parameswaran et al., 2012; Tagami et al., 2024), no studies have focused on optimizing the BAFF-based TCE structure. As a TNF superfamily ligand, BAFF relies on its native homotrimeric structure for its activity, which poses a significant challenge for its broader application and TCE development. The interactions between BAFF and its receptors critically depend on the unique homotrimeric configuration of BAFF, a structural feature that is challenging to replicate with conventional bivalent binding molecules, such as IgGs. To address this issue, Claus et al. introduced a 2 + 1-type design that successfully assembled trimeric 4-1BBL on an antibody backbone, demonstrating that clustering three 4-1BB receptors at the tumor site maximized 4-1BB agonism (Claus et al., 2019). Inspired by this approach, we initially developed a TriBAFF/CD3-IgG1 construct, but further investigation revealed its limited ability to redirect T cells. BAFF and other TNF superfamily ligands typically adopt a type II transmembrane protein architecture, where the C-terminus of the ligand ectodomain plays a pivotal role in receptor recognition. To overcome this limitation, we leveraged the inherent ability of BAFF to spontaneously form a stable trimeric structure by fusing antibodies to the N-terminus of its ligand ectodomains, resulting in a trivalent homotrimeric TCE construct. This construct, engineered to have the desired orientation, not only preserves the natural functionality of BAFF but also enables precise T-cell redirection against target tumor cells. Moreover, the flexible antibody format further enhanced the therapeutic potential of BAFF-based TCEs, offering a promising strategy to address antigen heterogeneity and improve efficacy when treating hematologic malignancies.

Antibody formats can be broadly classified as IgG-like subtypes, which include Fc regions, and non-IgG (or fragment) subtypes (Goebeler et al., 2024). IgG-like antibodies (such as TCEs) are known for their intrinsic stability, solubility, and prolonged half-life due to FcRn-mediated recycling (Brinkmann and Kontermann, 2017; Kontermann, 2009; Li et al., 2020). However, their structural complexity can lead to challenges such as chain mismatch, which have been addressed by innovative engineering strategies, such as KIH, CrossMab, and DuoBody (Schaefer et al., 2011; Surowka et al., 2021; Xu et al., 2015). In contrast, non-IgG constructs offer certain advantages, including enhanced tissue penetration and a reduced risk of unwanted immune activation (Goebeler et al., 2024), but their clinical utility is often limited by their shorter half-lives, which necessitates more frequent dosing, as observed with blinatumomab (Bargou et al., 2008). To improve the pharmacokinetic properties of BAFF-based TCEs, we explored strategies such as incorporating an inert Fc domain or a SA binding peptide (ABDCon), both of which successfully extend the half-life of the TCE while maintaining its biological activity (Arvedson et al., 2022; Einsele et al., 2020; Li et al., 2020). Nevertheless, the efficacy of the IgG-like isoform (Bivalent CD3-IgG1-TriBAFF) remains suboptimal compared with that of the Fab-structured TriBAFF/CD3/ABDCon both *in vitro* and *in vivo*. We propose that this discrepancy stems from the both trivalent architecture of TriBAFF/CD3/ABDCon and a spatial distance between the tumor antigen and the T-cell binding domain that is not excessive (Santich et al., 2020). In the case of the IS formed by the native T-cell receptor, the distance between the T cell and antigen presenting cell is reported to be ∼ 130–150 Å (Srivastava and Riddell, 2015; Wang et al., 2009). Previous studies have shown that this distance is crucial for the formation of a stable IS between T cells and tumor cells, which enhances T-cell activation and promotes tumor cell apoptosis by excluding the inhibitory phosphatase CD45 or CD148 (Calzada-Fraile et al., 2023; Chakraborty and Weiss, 2014; Li et al., 2017; Xiao et al., 2022). It is likely that the longer synapse resulting from the IgG format (Bivalent CD3-IgG1-TriBAFF) (∼ 70 Å longer than the TriBAFF/CD3/ABDCon TCE) is unable to sterically exclude these inhibitory molecules, resulting in less productive signal transduction. Compared with CD3-IgG1-TriBAFF, TriBAFF/CD3/ABDCon achieves superior T cell and tumor cell binding, leading to more robust activation and sustained signaling. Overall, our study emphasizes the importance of optimizing the antibody format according to the target and binding domain to achieve the ideal balance between efficacy and pharmacokinetics, ultimately maximizing the therapeutic potential of the TCE.

Current CAR-T cell and TCE therapies face significant challenges, including antigen escape, target downregulation, and tumor heterogeneity (Cappell and Kochenderfer, 2023; van de Donk and Zweegman, 2023), underscoring the urgent need for alternative combination therapies. Compared with the approved CD19-targeted therapies such as CAR-T cells and TCEs, TriBAFF/CD3/ABDCon exhibited markedly superior efficacy in a heterogeneous immune escape model of B-ALL and outperformed Blinatumomab and Tisagenlecleucel. Moreover, while the three BAFF targets of TriBAFF/CD3/ABDCon are relevant in a wide spectrum of B-cell malignancies, including leukemia, lymphoma, and myeloma, pro-B cells do not express these receptors (Wong et al., 2022), which could mitigate the risk of B-cell aplasia commonly associated with CD19-targeted therapies. On the other hand, although CAR-T cell therapy has revolutionized cancer treatment, its development is hindered by the time-consuming and resource-intensive individualized production processes, whereas TCEs offer distinct advantages as off-the-shelf products (Tapia-Galisteo et al., 2023; Trabolsi et al., 2024). In terms of safety, both the CAR-T and TCE strategies present similar toxicity profiles, including cytokine release syndrome (CRS) and immune effector cell-associated neurotoxicity syndrome (ICANS). Although direct comparisons within a single clinical trial are challenging, TCEs appear to offer a more favorable overall safety profile than CAR-T cell therapy does (Subklewe, 2021). In terms of efficacy, the superiority of TriBAFF/CD3/ABDCon over BAFF-based CAR-T cell therapy is evident, largely due to the preservation of the native BAFF ligand structure, as demonstrated in our previous report (Li et al., 2024). However, in a direct comparison with the sCAR-T approach, TriBAFF/CD3/ABDCon showed slightly reduced cytotoxicity at low drug concentrations, possible due to N-terminal steric hindrance affecting BAFF ligand accessibility. Despite this, TCE maintain important clinical advantages, including the elimination of exogenous T-cell infusion, no requirement for individualized manufacturing, and lower production costs. In contrast, CAR-T therapies remain more vulnerable to issues such as T-cell exhaustion. Nevertheless, these efforts alone are still not sufficient, and future studies comparing TriBAFF/CD3/ABDCon with more advanced therapies, such as trispecific TCEs, are essential to fully assess the potential of these novel strategies.

Our study indicates that developing trivalent TCEs that target T cells is feasible, although for most TCEs currently in clinical development, the focus has been on reducing the valency and affinity of CD3-specific antibodies to minimize nonspecific T-cell activation (Bacac et al., 2018; Zuch de Zafra et al., 2019). In our safety evaluation, the mice receiving the highest dose of TriBAFF/CD3/ABDCon (25 nmol/kg) tolerated the treatment well, with no toxic effects observed. Importantly, side effects are not necessarily eliminated with monovalent TCEs. For example, despite its monovalency, blinatumomab can still induce CRS and neurotoxicity (Klinger et al., 2012), but these effects can be mitigated with tocilizumab and steroid pretreatment (Friberg and Reese, 2017). An important pharmacokinetic consideration is the differential binding affinity of ABDCon to serum albumins across species. ABDCon exhibits higher affinity for human and rhesus monkey SA (*K*_D_ = 0.075 nmol/L and 0.061 nmol/L, respectively), but a substantially lower affinity for MSA (*K*_D_ = 3.22 nmol/L) (Jacobs et al., 2015). This weaker binding to MSA likely contributes to a shorter *in vivo* half-life in murine models. Furthermore, the accelerated clearance rate in mice may underestimate the potential toxicity associated with the trivalent CD3 design. Further studies, including those in nonhuman primates, are needed to fully assess the safety profile of this trivalent construct.

Although the optimal format, TriBAFF/CD3/ABDCon, showed promising therapeutic efficacy across leukemia, lymphoma, and myeloma, long-term follow-up revealed that eventual tumor relapse. Mechanistic investigations into Jeko-1 tumor recurrence identified both tumor antigen loss and T-cell exhaustion as major contributors. Similarly, in the ALL model, we observed a progressive decline in T cell counts during the later treatment stages and downregulation of target antigens in relapsed Nalm6 tumor cells. The results underscore that antigen escape and T-cell exhaustion remain significant barriers to the sustained efficacy of multispecific TCEs, whether in hematological malignancies or solid tumors. To address these challenges, future studies could explore combinatorial strategies such as co-administration of immune checkpoint inhibitors to reverse T-cell exhaustion, optimization of dose regimens to reduce the effects of continuous antigen exposure, and the use of protease inhibitors to prevent shedding of BAFFR, BCMA, and TACI, thereby enhancing receptor expression on the tumor cell surface. In conclusion, the limited efficacy of TCEs, particularly in solid tumors, is driven by a complex interplay of factors. Overcoming these limitations will require integrated strategies encompassing target selection refinement, enhancement of T-cell function, and modulation of the tumor microenvironment.

In conclusion, TriBAFF/CD3/ABDCon is a trifunctional TCE with an extended half-life that targets three key tumor antigens, BAFFR, BCMA, and TACI, via BAFF ligand incorporation to induce potent T-cell cytotoxic responses. Notably, TriBAFF/CD3/ABDCon not only effectively addresses CD19 immune escape but also outperforms conventional BAFF-based CAR-T cell therapy, positioning TriBAFF/CD3/ABDCon as a promising broad-spectrum treatment for B-cell malignancies. This study outlines a comprehensive framework for designing BAFF-based TCEs, which could significantly advance the field of T-cell redirection immunotherapy. Our innovative approach to generate trifunctional TCEs with optimized structural features offers great potential for enhancing natural ligand-based redirection therapies, with broad applications across various cancer types and nononcological indications.

## Supplementary data

Supplementary data is available at *Protein & Cell* online https://doi.org/10.1093/procel/pwaf054.

pwaf054_Supplementary_Data

## Data Availability

All the data that support the findings of this study are present in the paper and its Supplementary Information files.

## Abbreviations

ALL: acute lymphoblastic leukemia
ABDCon: albumin binding domain consensus sequence
BAFF: B-cell activating factor
BAFFR: BAFF-receptor
BCMA: B cell maturation antigen
BsAb: bispecific antibody
BiTE: bispecific T-cell engager
CAR-T: chimeric antigen receptor-T
CRISPR: clustered regularly interspaced short palindromic repeats
CLL: chronic lymphocytic leukemia
CRS: cytokine release syndrome
IS: immune synapse
ICANS: immune effector cell-associated neurotoxicity syndrome
KO: knockout
KIH: knob-into-hole
MM: multiple myeloma
MCL: mantle cell lymphoma; PKC-θ, protein kinase C-θ
SA: serum albumin
TACI: transmembrane activator and calcium modulator and cyclophilin ligand interactor
TCE: T-cell engager
TNF: tumor necrosis factor
