# Supplementary material for: BAFF-based trifunctional T-cell engagers trigger robust tumor immunity against B-cell malignancies
Source: Protein Cell. 2025 Jun 27;16(12):1017–34. doi: 10.1093/procel/pwaf054 (PMC12742853; doi:10.1093/procel/pwaf054)
Supplement: pwaf054_Supplementary_Data [file pwaf054_supplementary_data.pdf]

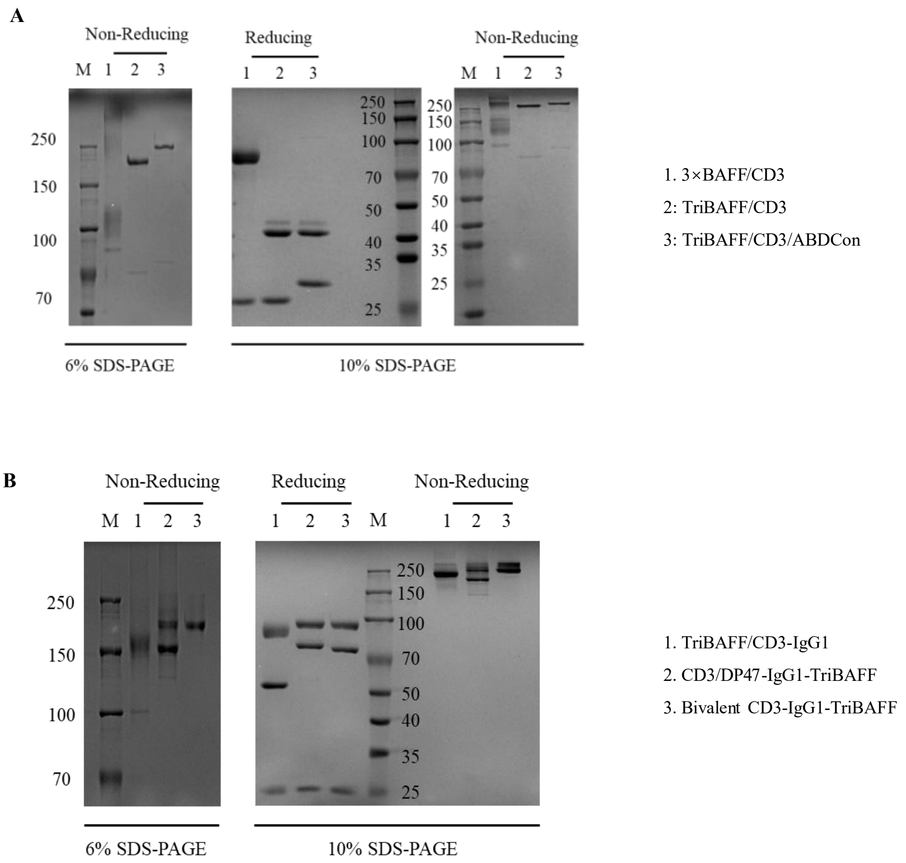

Figure S1. Characterization of different formats of BAFF-based TCEs.

(A) SDS-PAGE analysis of different formats of Fab-structured BAFF-based TCEs under non-reducing and reducing conditions. (B) SDS-PAGE analysis of different formats of IgG-structured BAFF-based TCEs under non-reducing and reducing conditions.

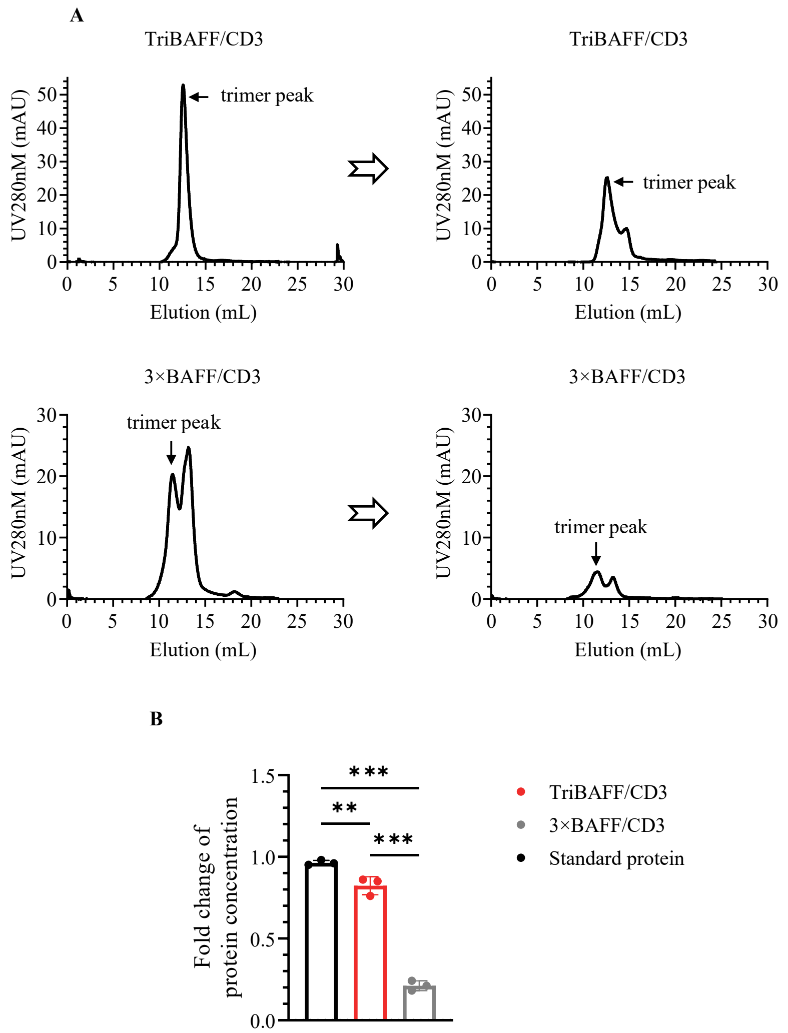

Figure S2. Protein stability analysis.

(A) SEC analysis of different formats of Fab-structured BAFF-based TCEs with different trimeric designs after five freeze-thaw cycles. (B) Quantitative analysis of protein concentration of different formats of Fab-structured BAFF-based TCEs with different trimeric designs after five freeze-thaw cycles. Standard protein: BSA.

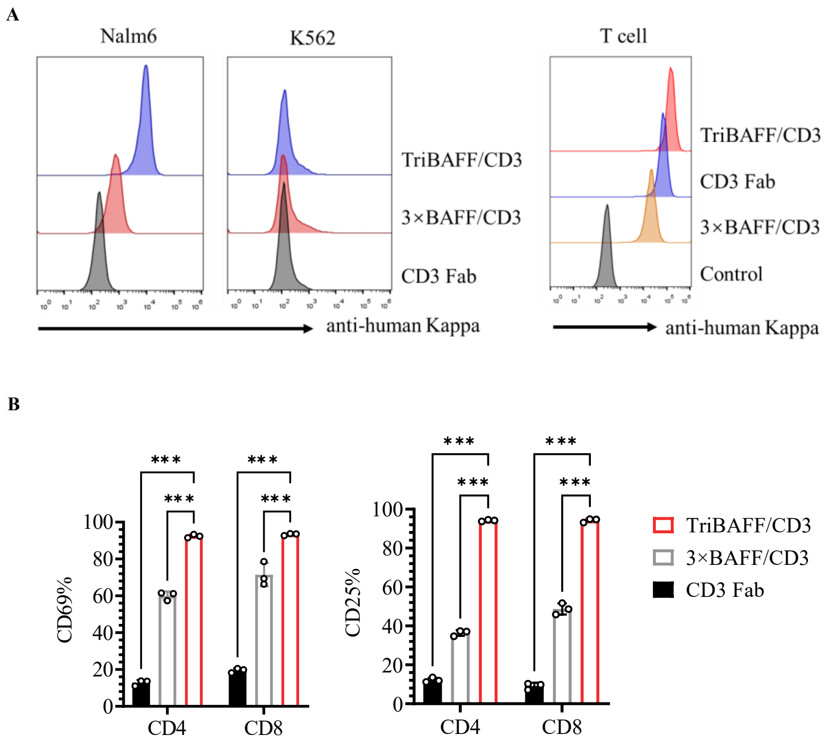

Figure S3. ***In vitro*** **binding and T cell activation activity of Fab-structured BAFF-based TCEs.** (A) Binding profiles of two fab-structured BAFF-based TCEs to tumor cells and human T cells were analyzed by flow cytometry. Assays included a CD3 Fab as a control. (B) Left: $CD69^+$ frequency on T cells after 20 hours of coculture with Nalm6 cells. Right: $CD25^+$ frequency on T cells after 96 hours of coculture with Nalm6 cells. Two-way ANOVA multiple comparisons in Dunnett correction were used to assess significance. Error bars represent means ± SD. *P < 0.05, **P < 0.01, and ***P < 0.001; ns indicates not significant (P ≥ 0.05).

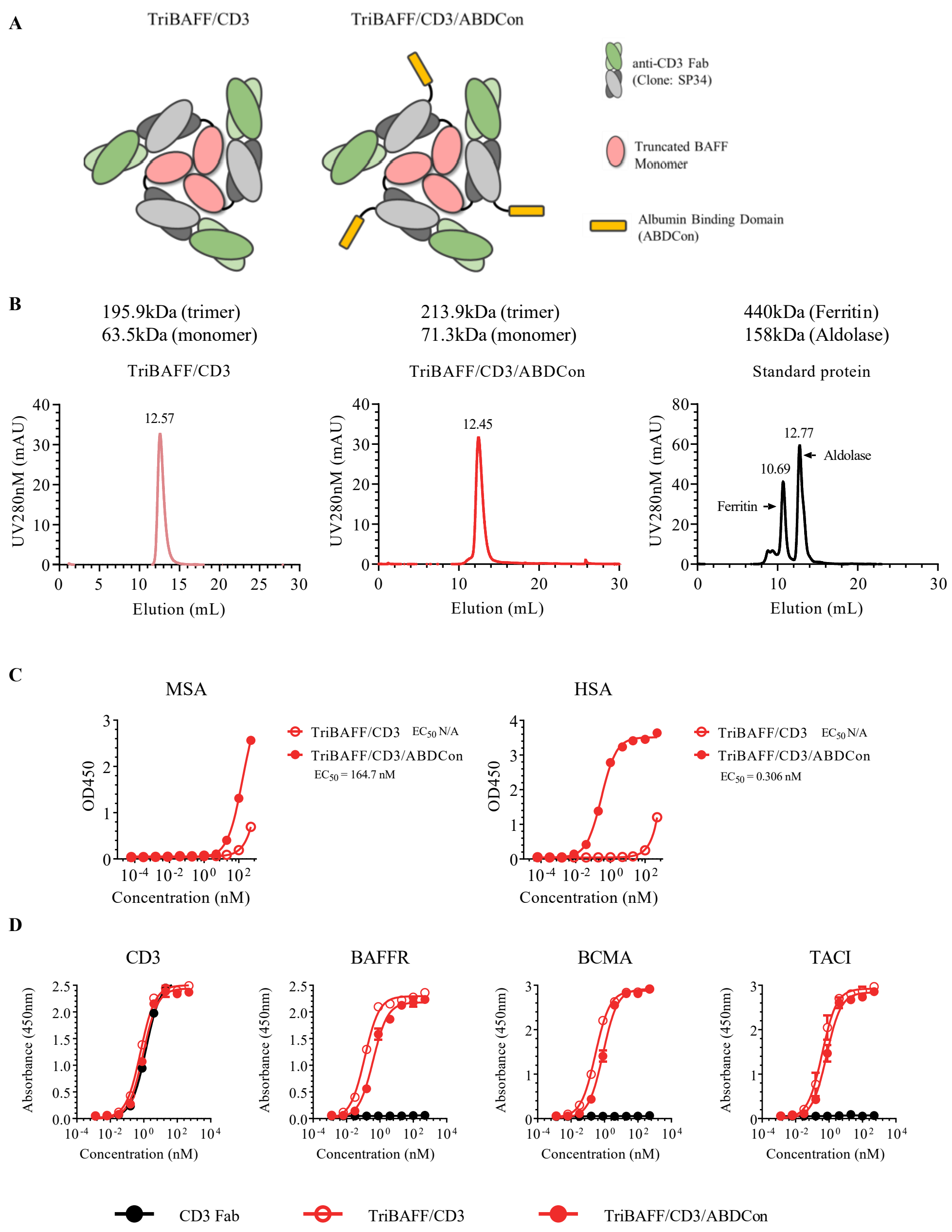

Figure S4. Preparation and characterization of trifunctional Fab-structured BAFF-based TCEs.

(A) Schematic of Fab-structured BAFF-based TCEs panel: TriBAFF/CD3 and TriBAFF/CD3/ABDCon. The green and grey domains represent anti-human CD3 Fab (Clone: SP34), the yellow domains represent albumin binding peptide (ABDCon), and the pink domains represent truncated BAFF ligands (134 aa-285 aa). (B) SEC analysis of TriBAFF/CD3, TriBAFF/CD3/ABDCon and standard proteins. Standard protein: ferritin and aldolase. (C) Binding profiles of two fab-structured BAFF-based TCEs to mouse serum albumin (MSA) and human serum albumin (HSA) were determined by ELISA. (D) Binding profiles of two fab-structured BAFF-based TCEs to antigens (CD3, BAFFR, BCMA, and TACI) were determined by ELISA. Assays included a CD3 Fab as a control. Error bars represent means ± SD.

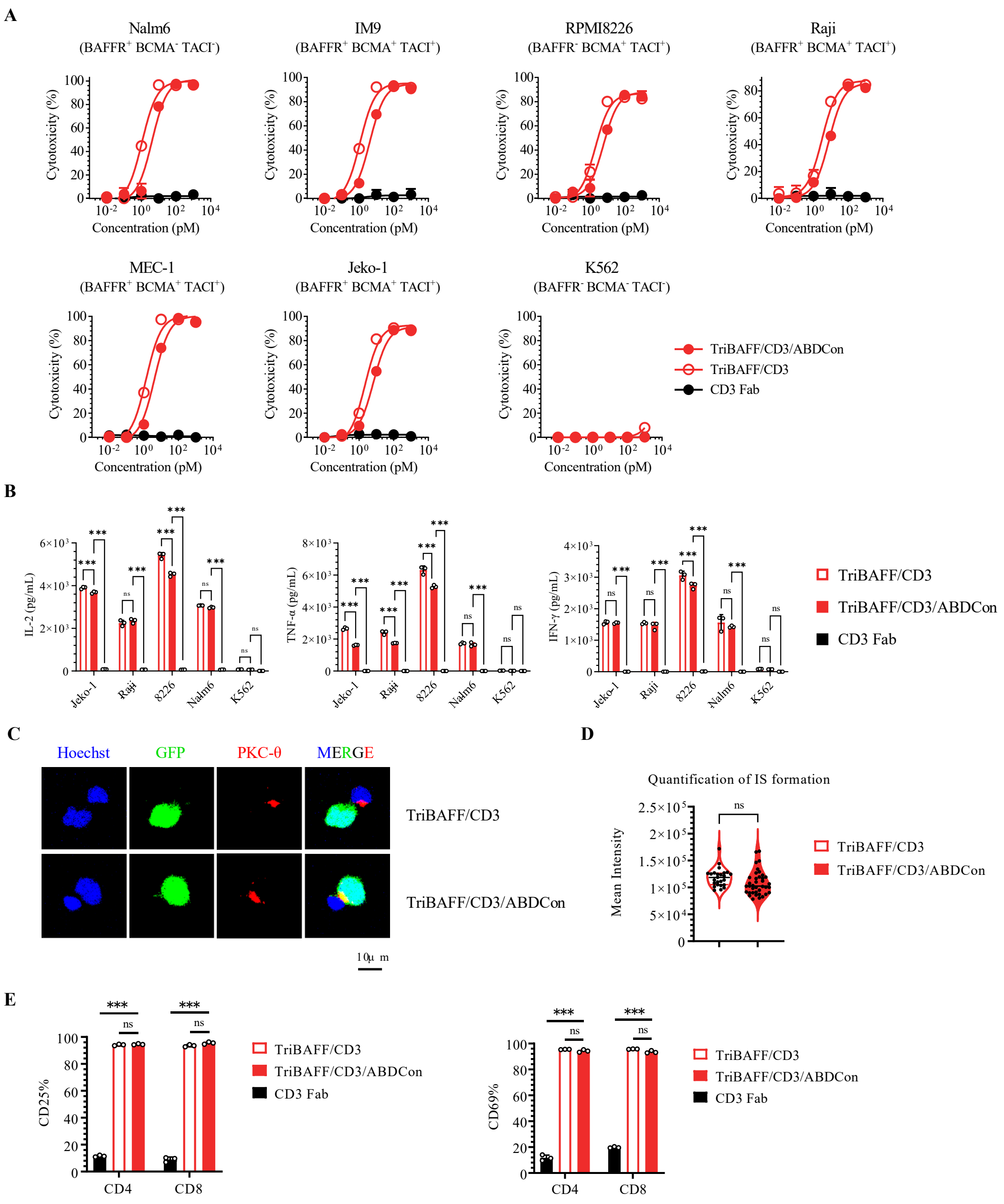

Figure S5. ***In vitro*** **functional validation of trifunctional Fab-structured BAFF-based TCEs.** (A) Cytotoxicity assays of different fab-structured BAFF-based TCEs were performed with T cells against the indicated target cells at an E:T ratio of 2:1 for 24 hours. Results are from one of two independent experiments. (B) Inflammatory cytokine release assays. Human T cells along with 1 nM corresponding fab-structured BAFF-based TCEs or CD3 Fab were co-cultured with the specific target cells for 24 hours at an E:T ratio of 1:1. Two-way ANOVA multiple comparisons in Dunnett correction were used to assess significance. (C) Representative confocal images of IS from three independent experiments. Human T cells were co-cultured with Nalm6-GFP cells in the presence of 1nM BAFF-based TCEs for 1 hour, and cell–cell conjugates were imaged at 100× oil objective magnification using a laser scanning confocal microscope (Nikon, A1R). Hoechst (blue), anti-PKC-θ (red), GFP (green) and merged images of all the stains are shown. Scale bar = 10 μm. (D) Statistical analysis of mean fluorescence intensity of PKC-θ at the IS in (C). P values were determined by paired two-tailed t-tests. (E) Right: $CD69^+$ frequency on T cells after 20 hours of coculture with

Nalm6 cells. Left: $CD25^+$ frequency on T cells after 96 hours of coculture with Nalm6 cells. Assays included a CD3 Fab as a control. Two-way ANOVA multiple comparisons in Dunnett correction were used to assess significance. Error bars represent means ± SD. *$P < 0.05$, **$P < 0.01$, and ***$P < 0.001$; ns indicates not significant ($P \geq 0.05$).

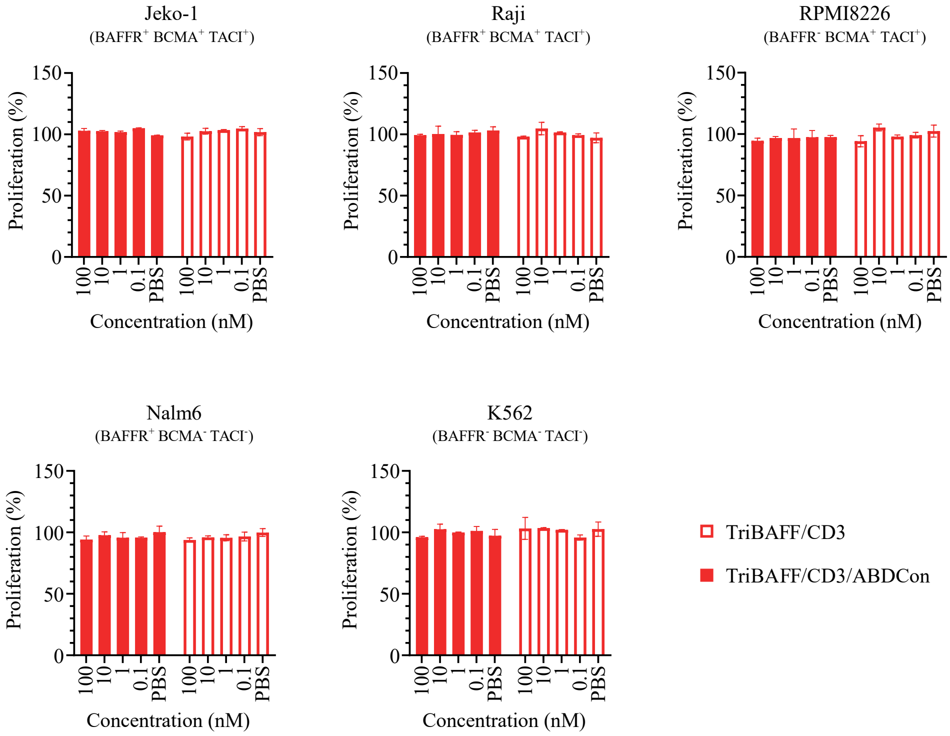

Figure S6 Tumor-promoting effect analysis of fab-structured BAFF-based TCEs.
Tumor cells were co-cultured with 1 nM concentrations of TriBAFF/CD3 or TriBAFF/CD3/ABDCon TCE for 5 days, followed by quantitative analysis of tumor cell counts using flow cytometry. Data are shown as means ± SD.

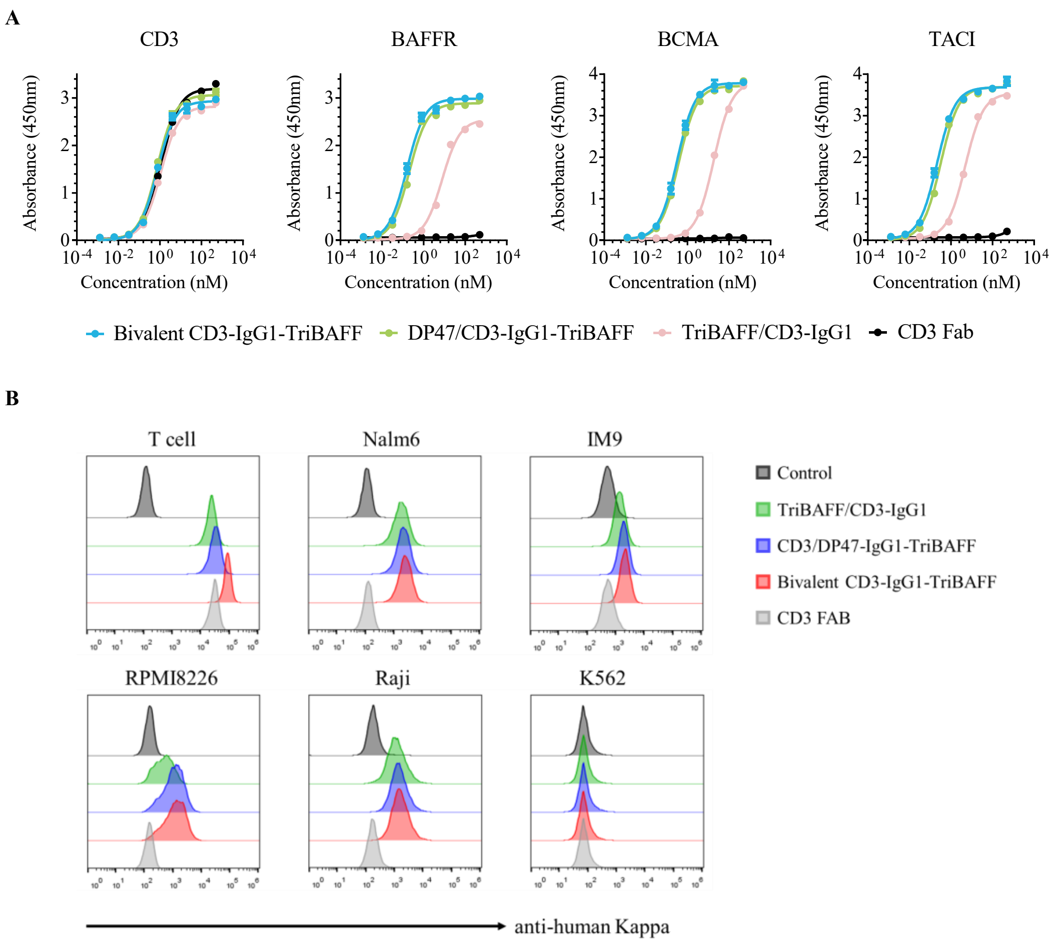

Figure S7. *In vitro* binding activity of IgG-structured BAFF-based TCEs.

(A) Binding profiles of three IgG-structured BAFF-based TCEs to antigens (CD3, BAFFR, BCMA, and TACI) were determined by ELISA. Assays included a CD3 Fab as a control. (B) Binding profiles of three IgG-structured BAFF-based TCEs to tumor cells and human T cells were analyzed by flow cytometry. Assays included a CD3 Fab as a control. Error bars represent means ± SD.

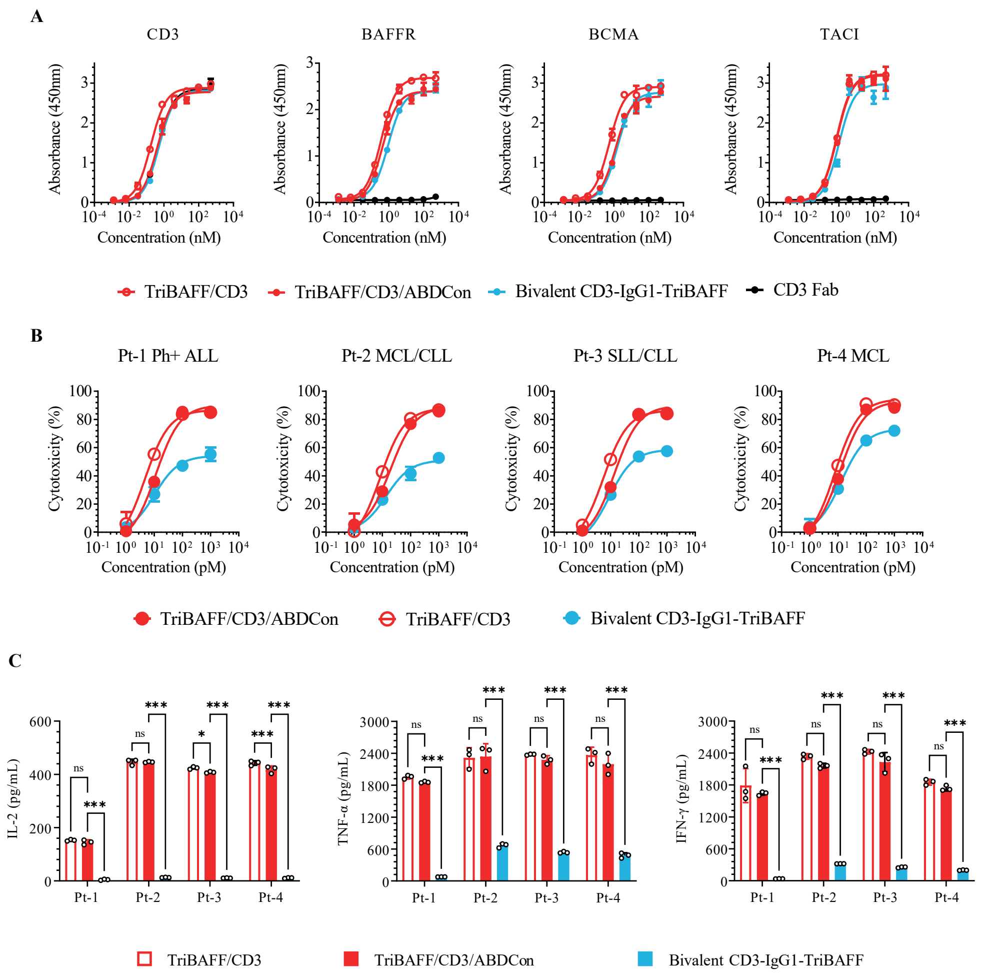

Figure S8. *In vitro* binding activity and primary cell cytotoxicity of the optimal trifunctional BAFF-based TCEs.

(A) Binding profiles of different BAFF-based TCEs to antigens (CD3, BAFFR, BCMA, and TACI) were determined by ELISA. Assays included a CD3 Fab as a control. (B) Cytotoxicity assays of different BAFF-based TCEs were performed with T cells against the indicated primary tumor cells at an E:T ratio of 2:1 for 24 hours. Results are from one of two independent experiments. (C) Inflammatory cytokine release assays. Human T cells along with 1 nM corresponding BAFF-based TCEs or CD3 Fab were co-cultured with the specific primary tumor cells for 24 hours at an E:T ratio of 1:1. Two-way ANOVA multiple comparisons in Dunnett correction were used to assess significance. Error bars represent means ± SD. *$P < 0.05$, **$P < 0.01$, and ***$P < 0.001$; ns indicates not significant ($P \geq 0.05$).

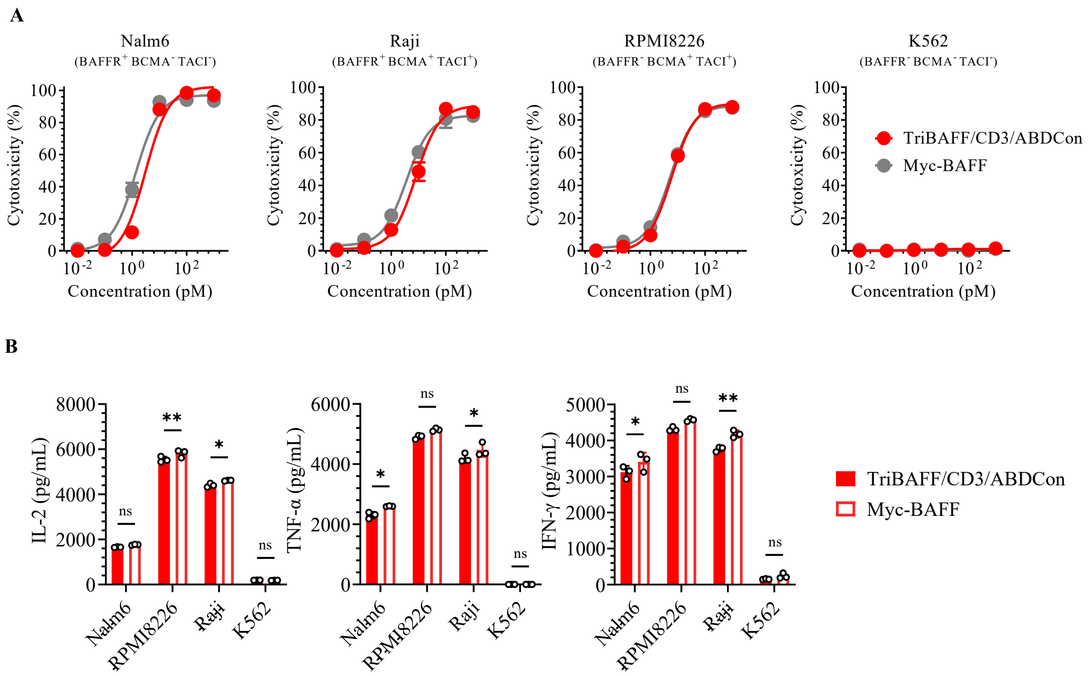

Figure S9. Comparison of TriBAFF/CD3/ABDCon-mediated T cells and Myc-BAFF-mediated sCAR-T cells.

(A) Cytotoxicity assays of TriBAFF/CD3/ABDCon and Myc-BAFF were performed with T cells or 9E10-IgG4m sCAR-T cells against the indicated target cells at an E:T ratio of 2:1 for 24 hours. Results are from one of more than two independent experiments. Error bars represent SD. (B) Inflammatory cytokine release assay. T cells along with 1 nM TriBAFF/CD3/ABDCon or 9E10-IgG4m sCAR-T cells along with 1 nM Myc-BAFF were co-cultured with the specific target cells for 24 hours at an E:T ratio of 1:1. Two-way ANOVA multiple comparisons in Dunnett correction were used to assess significance. Data are shown as means ± SD. *$P < 0.05$, **$P < 0.01$, and ***$P < 0.001$; ns, not significant (⩾0.05).

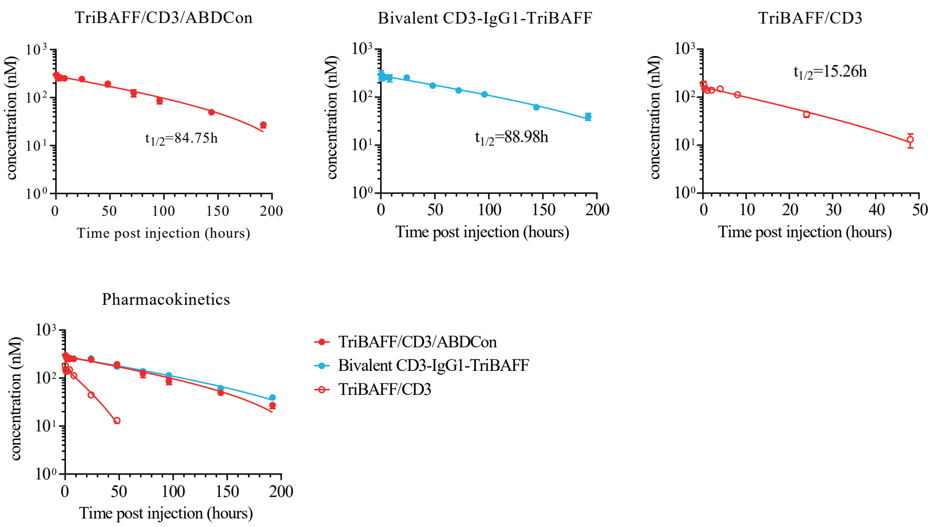

Figure S10. Pharmacokinetic of BAFF-based TCEs.

Half-life of TriBAFF/CD3, TriBAFF/CD3/ABDCon and Bivalent CD3-IgG1-TriBAFF after single i.v. administration at 25 nmol/kg in female BALB/c mice (6–8 weeks). The concentrations of different BAFF-based TCEs in peripheral blood samples were extrapolated from a standard curve. Half-life parameters were analyzed via GraphPad Prism analysis software. The means ± SD are shown for each timepoint.

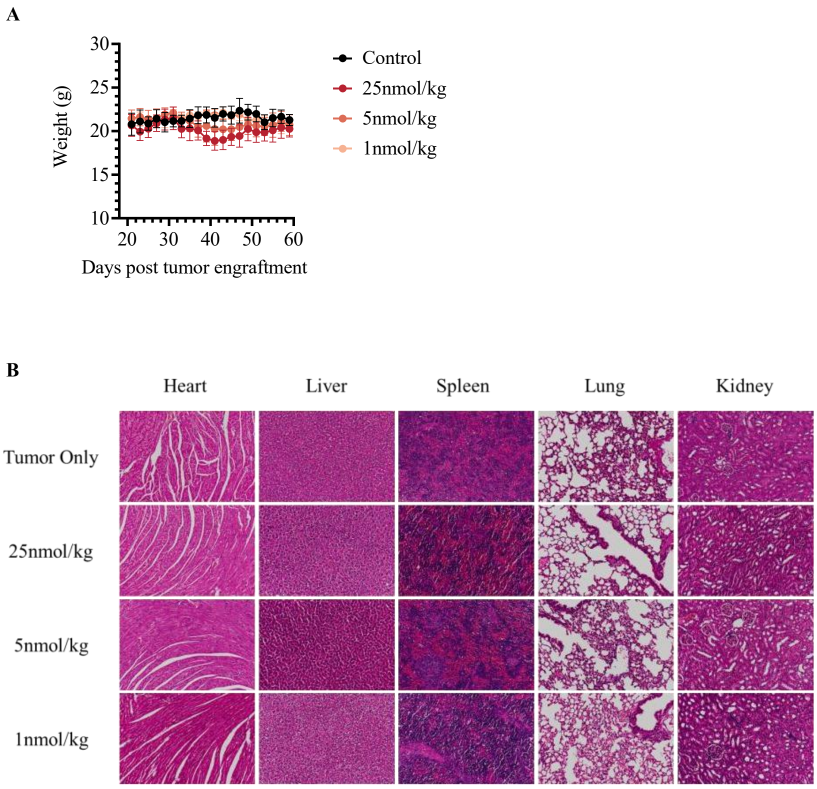

Figure S11. Safety analysis of trifunctional BAFF-based TCEs in MM model.

(A) Mice body weight changes in MM model during TCE administrations. Error bars represent mean ± SEM.(B) Histological sections of tissues from mice treated with PBS or 25, 5, 1 nmol/kg TriBAFF/CD3/ABDCon are shown. Heart, liver, spleen, lung and Kidney were harvested from euthanized mice 43 days post-inoculation, and tissue sections were stained with hematoxylin and eosin.

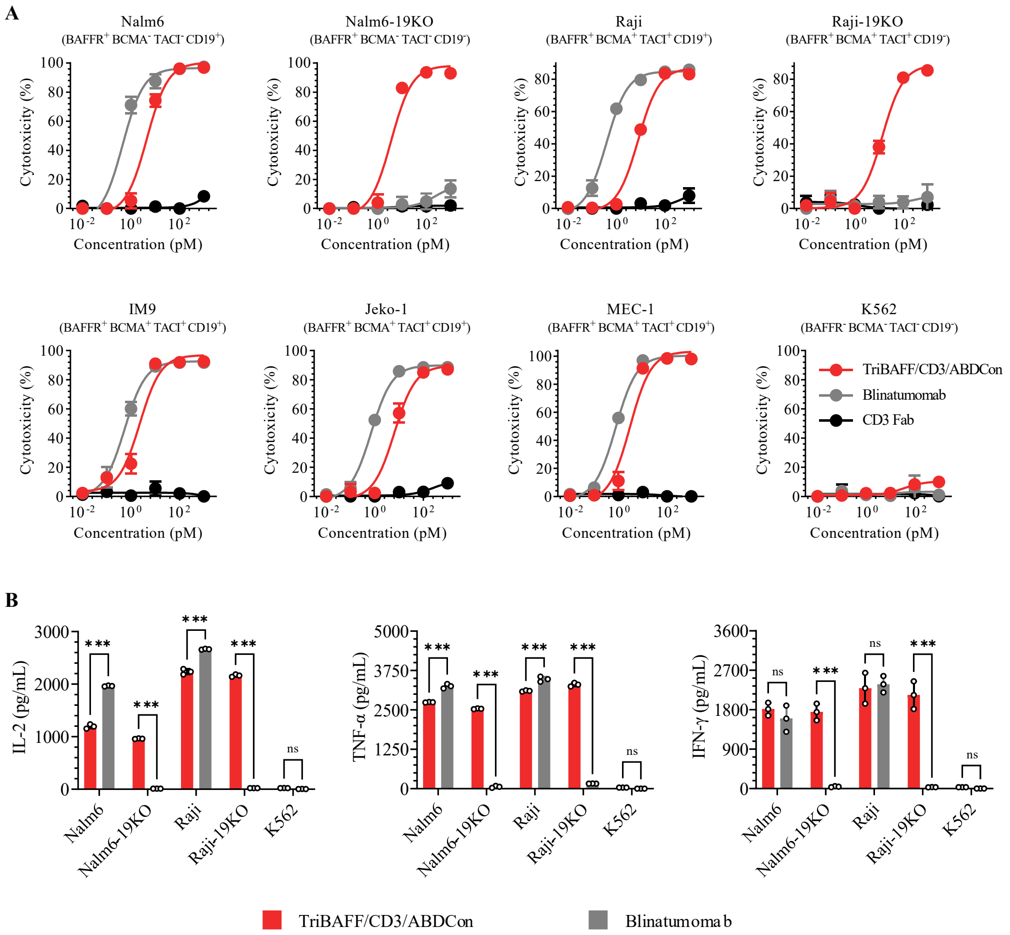

Figure S12. *In vitro* comparison of TriBAFF/CD3/ABDCon to blinatumomab.

(A) Cytotoxicity assays of TriBAFF/CD3/ABDCon and blinatumomab were performed with T cells against the indicated target cells at an E:T ratio of 2:1 for 24 hours. Results are from one of two independent experiments. (B) Inflammatory cytokine release assays. Human T cells along with 1 nM corresponding BAFF-based TCEs were co-cultured with the specific target cells for 24 hours at an E:T ratio of 1:1. Two-way ANOVA multiple comparisons in Dunnett correction were used to assess significance. Error bars represent means ± SD.*$P < 0.05$, **$P < 0.01$, and ***$P < 0.001$; ns indicates not significant ($P \geq 0.05$).

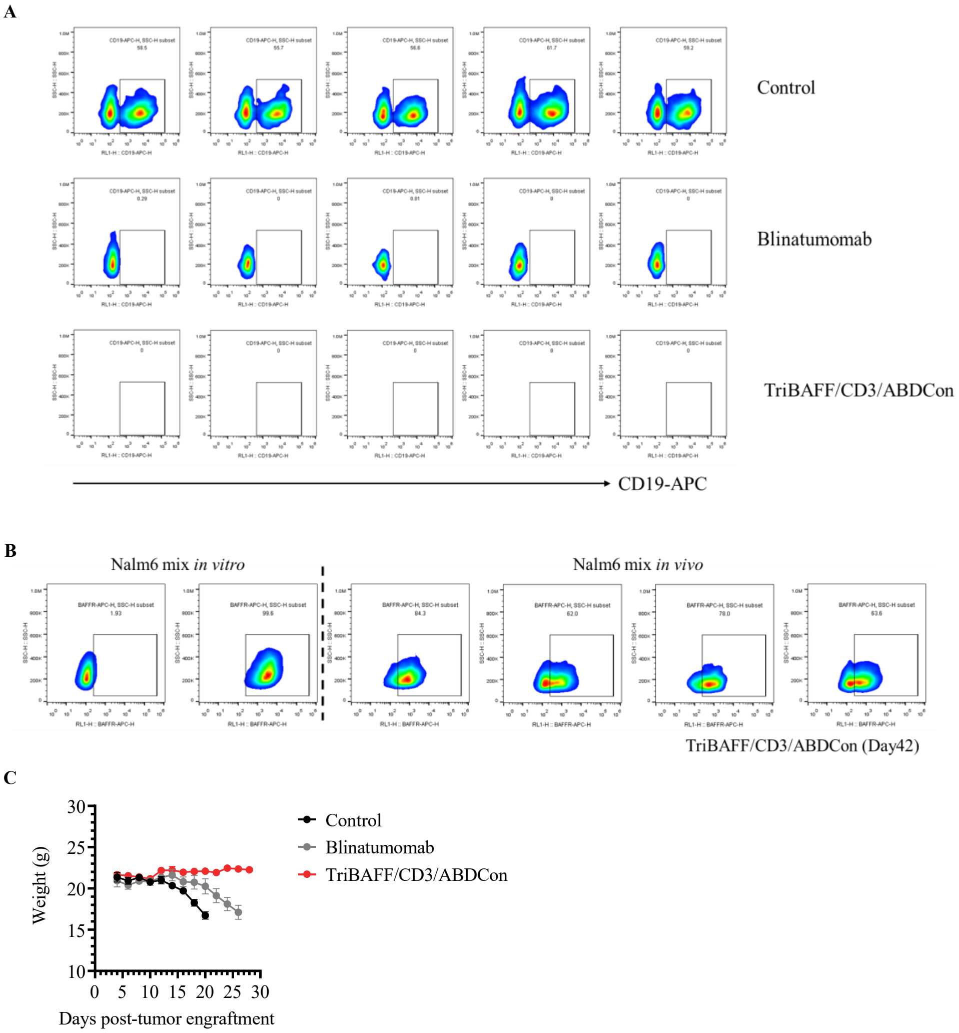

Figure S13. Tumor cell antigens detection and body weight monitoring to compare TriBAFF/CD3/ABDCon with blinatumomab in the heterogeneous B-ALL model.

(A) CD19 expression levels on the tumor cells were analyzed on day 20 of the experiment using flow cytometry. The tumor cells were identified as $GFP^{+}CD19^{+}$ or $GFP^{+}CD19^{-}$. (B) BAFFR expression on the tumor cell surface was analyzed on day 42 in mice where tumor relapse was observed after treatment with TriBAFF/CD3/ABDCon. BAFFR expression of Nalm6 cells cultured in vitro was used as the flow cytometry gating strategy. (C) Changes in the body weights of the mice during blinatumomab or TriBAFF/CD3/ABDCon administrations. Error bars represent means ± SEM.

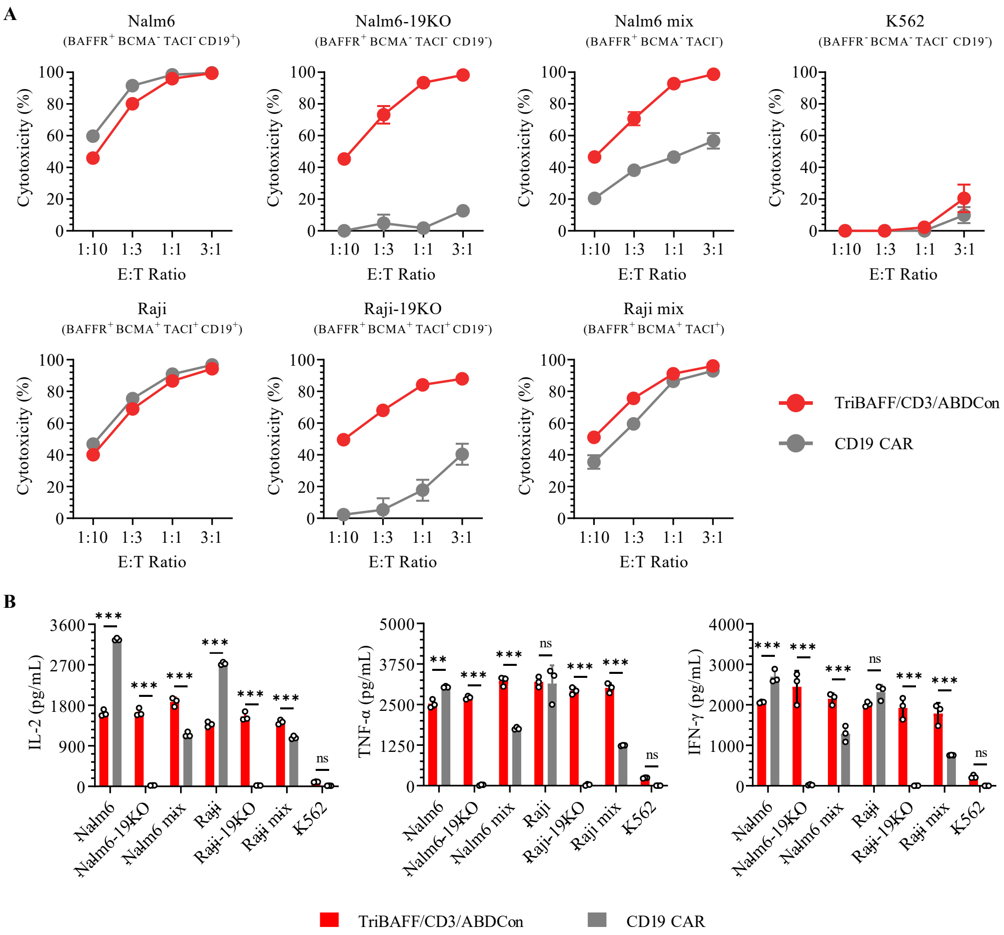

Figure S14. *In vitro* comparison of TriBAFF/CD3/ABDCon to CD19 CAR.

(A) Cytotoxicity assays of human T cells along with 1 nM TriBAFF/CD3/ABDCon or CD19 CAR were performed against the indicated target cells at virous E:T ratios for 24 hours. (B) Inflammatory cytokine release assays. Human T cells along with 1 nM TriBAFF/CD3/ABDCon or CD19 CAR-T cells were co-cultured with the specific target cells for 24 hours at an E:T ratio of 1:1. Two-way ANOVA multiple comparisons in Dunnett correction were used to assess significance. Results are from one of two independent experiments. Data are shown as means ± SD. *P < 0.05, **P < 0.01, and ***P < 0.001; ns, not significant (⩾0.05).

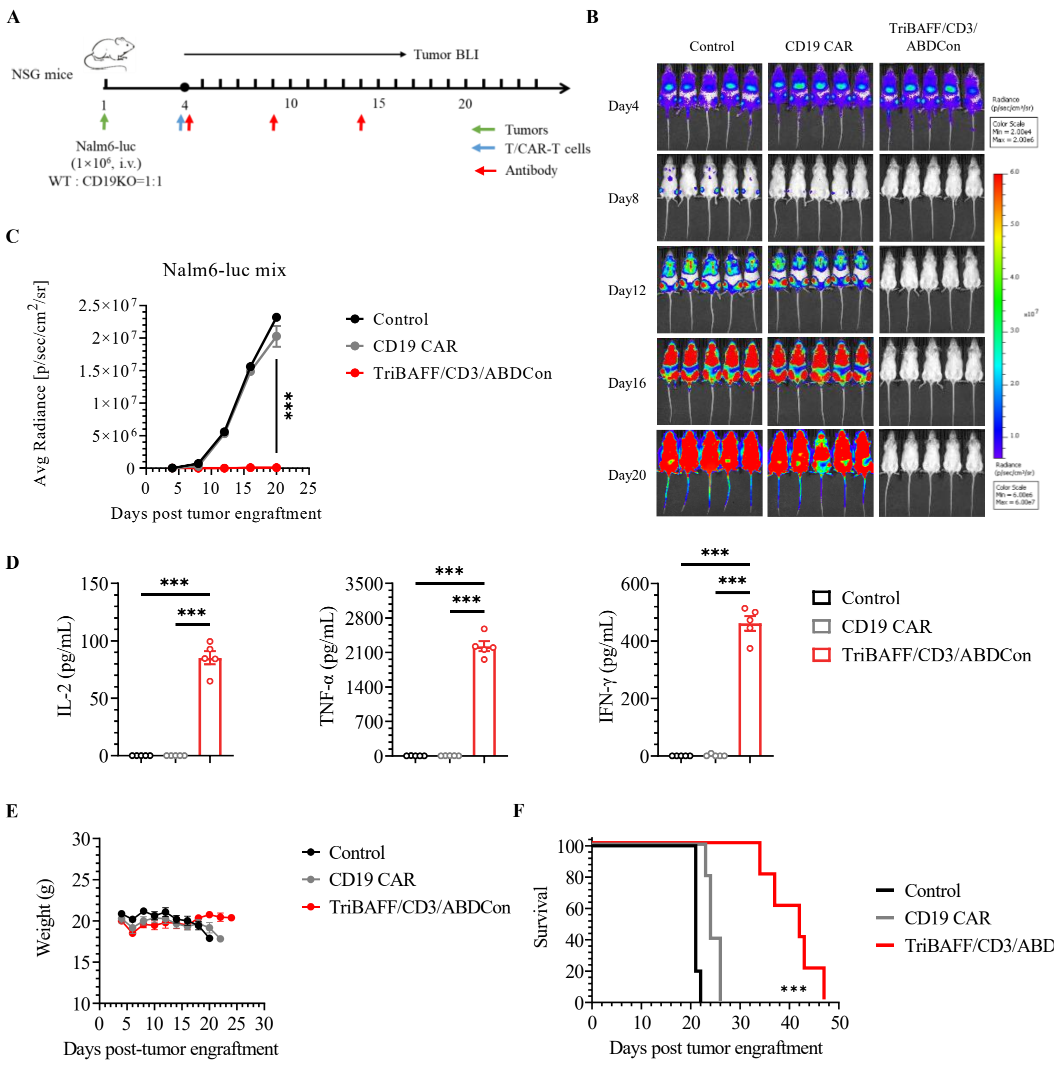

Figure S15. *In vivo* comparison of TriBAFF/CD3/ABDCon to CD19 CAR.

(A) Timeline of the *in vivo* experiments. The red triangles represent TriBAFF/CD3/ABDCon administration. Consistent results were obtained from two independent experiments (n = 5 mice). (B) Representative bioluminescence images of mice subjected to different treatments. The colors represent the luminescence intensity (red, highest; blue, lowest). (C) Quantification of the average luminescence intensity (p/s/cm^2/sr). Two-way ANOVA multiple comparisons in Dunnett correction were used to assess the significance, comparing TriBAFF/CD3/ABDCon with CD19 CAR. (D) Evaluation of serum inflammatory cytokine release by ELISA 2 hours after TCE administration. One-way ANOVA multiple comparisons in Dunnett correction were used to assess the significance. (E) Changes in the body weights of the mice. (F) Survival curves of the mice subjected to the indicated treatments compared using the log-rank (Mantel–Cox) test. Error bars represent means ± SEM. *P < 0.05, **P < 0.01, and ***P < 0.001; ns indicates not significant (P ≥ 0.05).

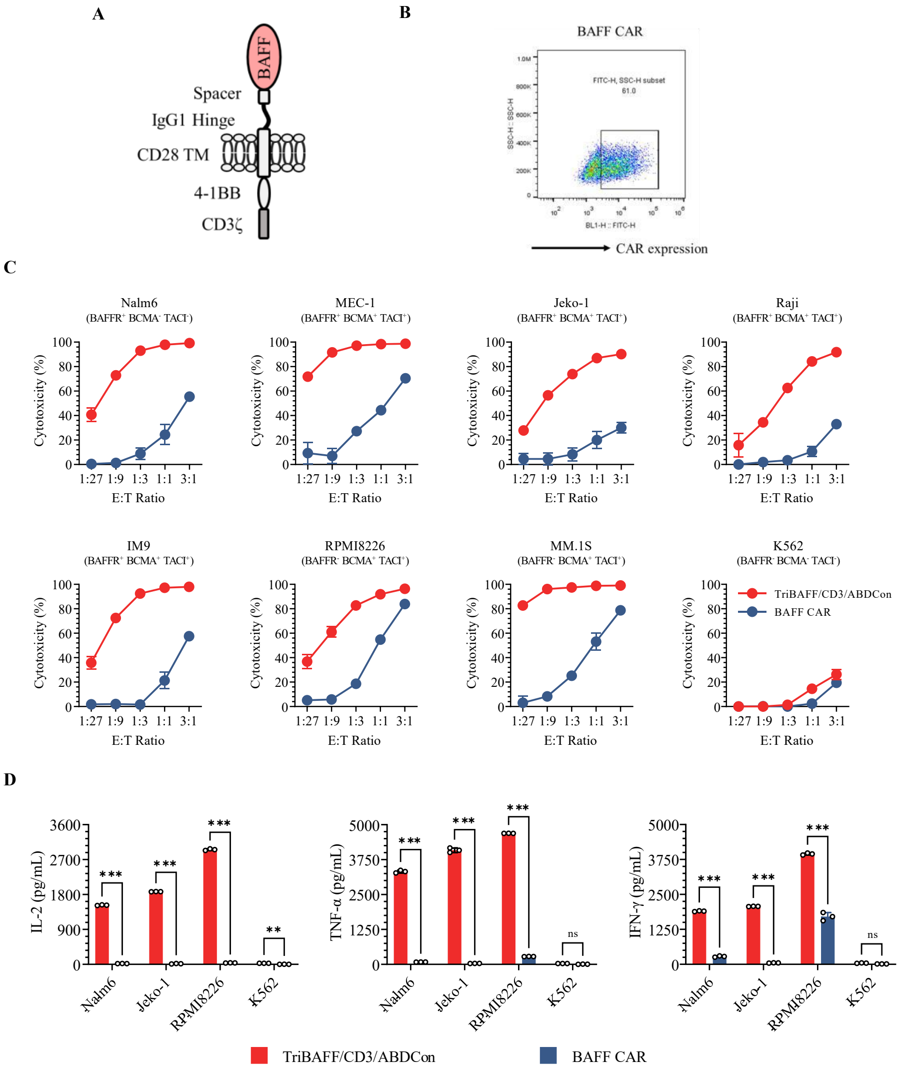

Figure S16. ***In vitro*** **comparison of TriBAFF/CD3/ABDCon to BAFF-based CAR.**

(A) The schematic of the BAFF-based CAR structure, termed BAFF CAR. BAFF CAR used the extracellular domain of BAFF ligands as the target moiety, featuring a Myc tag at the N-terminus and incorporating the IgG1 hinge region and CD28 transmembrane domain. (B) Representative flow cytometry plots depicting the expression of BAFF CAR. (C) Cytotoxicity assays of TriBAFF/CD3/ABDCon and BAFF CAR were performed against the indicated target cells at virous E:T ratios for 24 hours. Results are from one of two independent experiments. (D) Inflammatory cytokine release assays. Human T cells along with 1 nM corresponding TriBAFF/CD3/ABDCon or BAFF CAR-T cells were co-cultured with the specific target cells for 24 hours at an E:T ratio of 1:1. Two-way ANOVA multiple comparisons in Dunnett correction were used to assess significance. Error bars represent means ± SD.*P < 0.05, **P < 0.01, and ***P < 0.001; ns indicates not significant (P ≥ 0.05).

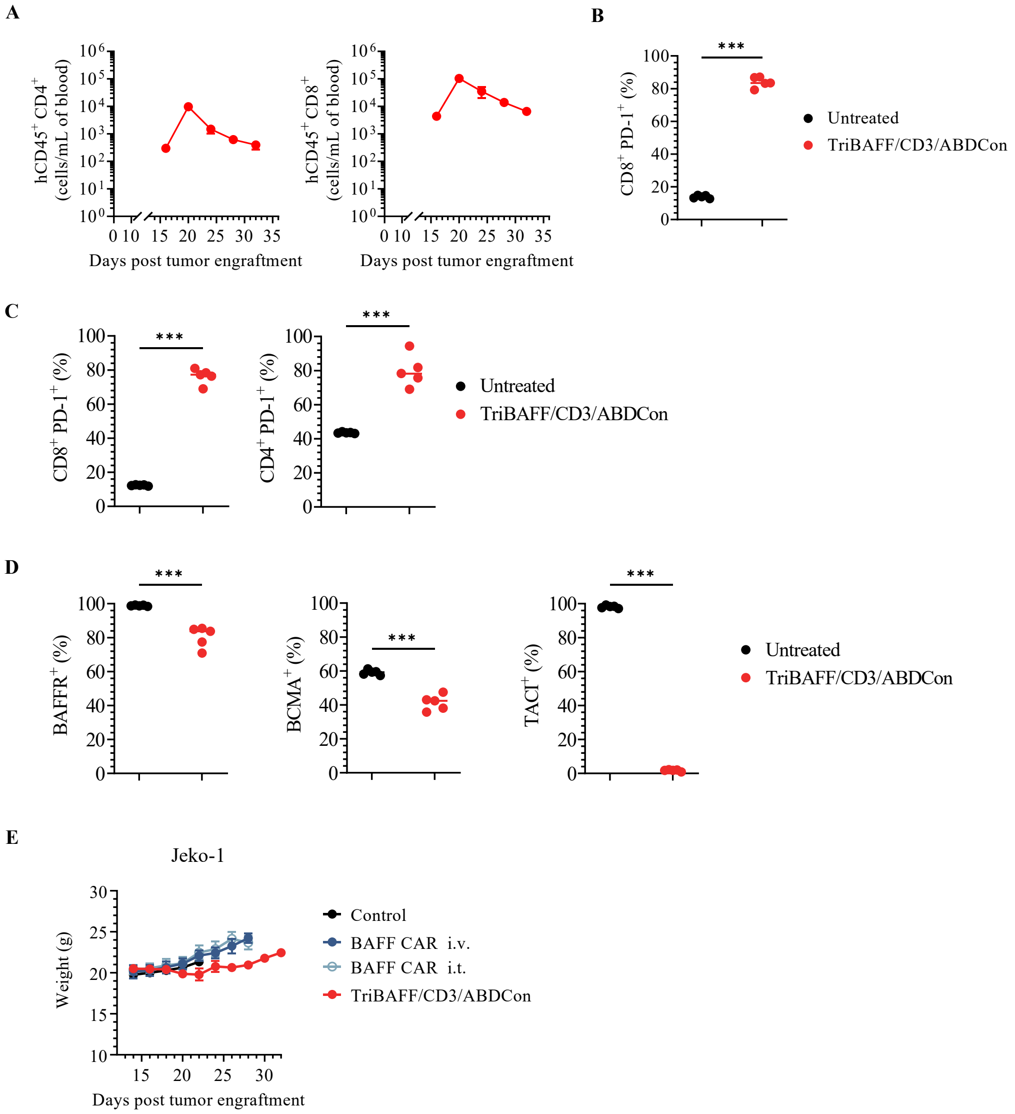

Figure S17. Additional data of TriBAFF/CD3/ABDCon compared to BAFF-based CAR in Jeko-1 model.

(A) Assessment of the presence of persistent human CD4$^+$ (hCD45$^+$ CD4$^+$, left) and CD8$^+$ (hCD45$^+$ CD8$^+$, right) T cells in the peripheral blood of mice treated with TriBAFF/CD3/ABDCon by flow cytometry. (B) On day 32, the expression levels of PD-1 on the surface of CD8$^+$ T cells in the peripheral blood of mice treated with TriBAFF/CD3/ABDCon were analyzed by flow cytometry. Untreated: in vitro-activated T cells from the same batch. One-way ANOVA multiple comparisons in Dunnett correction were used to assess the significance. (C) On day 32, the expression levels of PD-1 on the surface of human tumor-infiltrating CD8$^+$ (hCD45$^+$ CD8$^+$, left) and CD4$^+$ (hCD45$^+$ CD4$^+$, right) T cells in the of mice treated with TriBAFF/CD3/ABDCon were analyzed by flow cytometry. Untreated: in vitro-activated T cells from the same batch. One-way ANOVA multiple comparisons in Dunnett correction were used to assess the significance. (D) On day 32, the BAFFR, BCMA and TACI antigen expression levels on the surface of relapse tumor cells in the of mice treated with TriBAFF/CD3/ABDCon were analyzed by flow cytometry. Untreated: in vitro cultured Jeko-1 cells. One-way ANOVA multiple comparisons in Dunnett correction were used to assess the significance. (E) Mice body weights changes in Jeko-1 model during TCE administration. Error bars represent SEM. *P < 0.05, **P < 0.01, and ***P < 0.001; ns, not significant (≥0.05).

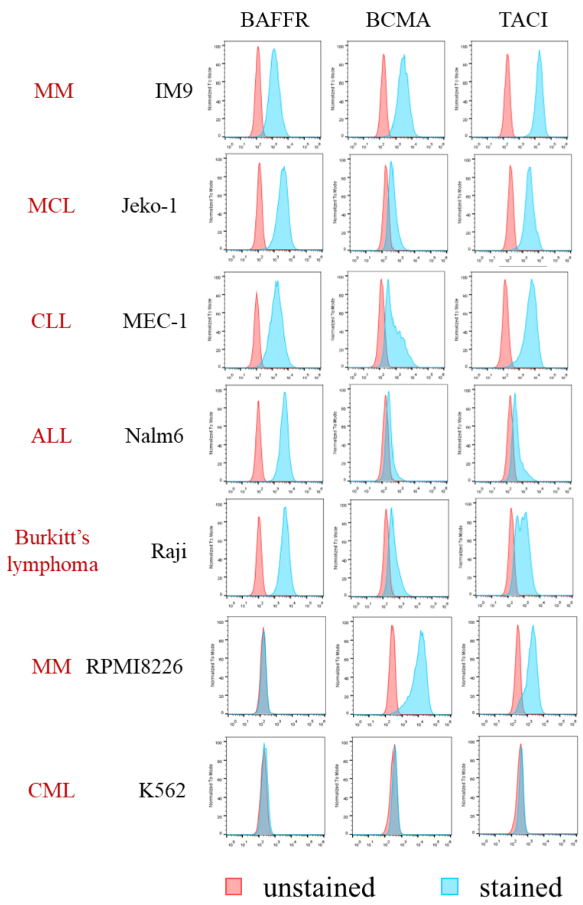

Figure S18. Comprehensive expression of BAFFR, BCMA, and TACI antigens in B-cell malignancies.

Representative flow cytometry plots illustrating the antigens in various B-cell malignancies. Surface expression of BAFFR, BCMA and TACI antigens was evaluated using flow cytometry with APC conjugated anti-human BAFFR antibody, PE-conjugated anti-human BCMA antibody and PE conjugated anti-human TACI antibody. Data are representative of five independent experiments.

Table S1. Protein yields of each construct.

Construct	Yield (mg/L)
3×BAFF/CD3	3
TriBAFF/CD3	12.5
TriBAFF/CD3/ABDCon	10
TriBAFF/CD3-IgG1	15
CD3/DP47-IgG1-TriBAFF	40
Bivalent CD3-IgG1-TriBAFF	50
